# A peach esterase/lipase mediates lipid remodeling as a protective mechanism against chilling injury

**DOI:** 10.1093/plphys/kiag705

**Published:** 2026-09-21

**Authors:** Mengfei Liu, Xinyue Chen, Wanyi Yang, Shaojie Ai, Lulu Wu, Yanna Shi, Guojian Hu, Kui Lin-Wang, Andrew C Allan, Kunsong Chen, Changjie Xu

**Affiliations:** College of Agriculture & Biotechnology, Zhejiang University, Zijingang Campus, Hangzhou 310058, China; College of Agriculture & Biotechnology, Zhejiang University, Zijingang Campus, Hangzhou 310058, China; College of Agriculture & Biotechnology, Zhejiang University, Zijingang Campus, Hangzhou 310058, China; College of Agriculture & Biotechnology, Zhejiang University, Zijingang Campus, Hangzhou 310058, China; Cuisine Science Key Laboratory of Sichuan Province, Sichuan Tourism University, Chengdu 610100, China; College of Agriculture & Biotechnology, Zhejiang University, Zijingang Campus, Hangzhou 310058, China; College of Agriculture & Biotechnology, Zhejiang University, Zijingang Campus, Hangzhou 310058, China; College of Agriculture & Biotechnology, Zhejiang University, Zijingang Campus, Hangzhou 310058, China; New Zealand Institute for Bioeconomy Science Limited, Private Bag 92169, Auckland Mail Centre, Auckland 1142, New Zealand; New Zealand Institute for Bioeconomy Science Limited, Private Bag 92169, Auckland Mail Centre, Auckland 1142, New Zealand; School of Biological Sciences, University of Auckland, Private Bag 92019, Auckland 1142, New Zealand; College of Agriculture & Biotechnology, Zhejiang University, Zijingang Campus, Hangzhou 310058, China; Zhejiang Key Laboratory of Horticultural Crop Quality Improvement, Zhejiang University, Zijingang Campus, Hangzhou 310058, China; College of Agriculture & Biotechnology, Zhejiang University, Zijingang Campus, Hangzhou 310058, China; Key Laboratory of Horticultural Plant Growth and Development, Ministry of Agriculture and Rural Affairs, Zhejiang-University, Zijingang Campus, Hangzhou 310058, China

## Abstract

Peach fruit is highly susceptible to chilling injury, which induces internal browning during long-term low-temperature storage. Internal browning was found to occur only at intermediate temperatures (5, 8, and 12 °C), but not at either lower (0 °C) or higher (16 °C) temperatures. Proteomic analysis of peach fruit stored at different temperatures for various durations was performed, and a GDSL esterase/lipase, PpGELP18, a protein with in vitro esterase/lipase activity, was identified. Transgenic tobacco (*Nicotiana tabacum*) overexpressing *PpGELP18* demonstrated enhanced chilling tolerance. Lipidomic analysis of transgenic tobacco (*N. tabacum*) leaves and peach fruit revealed that PpGELP18 is involved in intracellular lipid remodeling, particularly in the accumulation of membrane phospholipids. Elevated levels of unsaturated phosphatidylcholines and phosphatidylethanolamines, along with a decrease in lysophospholipids and diacylglycerols, were observed in transgenic tobacco (*N. tabacum*). In contrast, a decrease in phospholipids and an increase in diacylglycerols occurred in peach fruit at chilling-injury-inducing temperatures and during the late storage period at 5 °C, accompanied by internal browning. Ultrastructural examination confirmed that chilling-injury-associated internal browning was accompanied by damage to the plasma membrane and tonoplast. These results demonstrate that elevated PpGELP18 confers chilling tolerance in both peach fruit and tobacco (*N. tabacum*) plants by modulating lipid composition.

## Introduction

Low-temperature stress is one of the primary abiotic stresses that adversely affect plant growth, development, and physiological metabolism. Freezing temperatures below 0 °C can induce intracellular ice formation and compromise membrane integrity (Yamazaki et al. 2008). Although temperatures above 0 °C do not cause mechanical damage from ice crystal formation, prolonged exposure to non-freezing chilling stress can still disrupt physiological processes. The severity of chilling injury (CI) largely depends on the plant genotype and the duration of exposure to unfavorable temperatures (Jung et al. 2023). Analogously, during the postharvest storage of fruits and vegetables, inappropriate low temperatures often induce postharvest CI, a common physiological disorder that significantly compromises commodity quality and shelf life. Notably, peach (*Prunus persica*) fruit is particularly susceptible to CI during storage and frequently exhibits typical symptoms including mealiness, internal browning (IB), and loss of flavor, all of which substantially reduce its commercial value (Lurie and Crisosto 2005; Fruk et al. 2014; Lurie 2021; Rodrigues et al. 2024).

The mechanisms by which plants adapt to low-temperature stress are orchestrated through the perception of low temperatures, the expression of cold-responsive genes, and subsequent metabolic regulation (Ding et al. 2024b). Among these, the regulation of lipid metabolism has also been recognized as associated with cold tolerance in plants as diverse as Arabidopsis (*Arabidopsis thaliana*) and maize (*Zea mays*) (Welti et al. 2002; Gao et al. 2024). Lipids are essential structural and functional constituents of plant cells, encompassing diverse classes such as glycerolipids, sphingolipids, and sterols. In Arabidopsis leaves, glycerolipids represent the most abundant lipid category, while sphingolipids and sterols constitute minor fractions (Browse and Somerville 1994). Glycerolipids can be categorized into 3 main subtypes: storage lipids, glycolipids, and phospholipids (PLs). Among these, PLs serve as fundamental structural components of biological membranes (Harayama and Riezman 2018). The structure and lipid composition of these membranes are profoundly affected by low-temperature stress, with alterations including the modulation of lipid unsaturation to maintain membrane fluidity and extensive lipid remodeling, which collectively facilitate cellular adaptation to environmental changes (Peppino Margutti et al. 2018; Shomo et al. 2024). With the advancement and application of lipidomics technology, lipid remodeling has been reported to occur during the occurence of CI in maize, barley (*Hordeum vulgare*), rice (*Oryza sativa*), and Arabidopsis plants (Zheng et al. 2016; Peppino Margutti et al. 2018; Gao et al. 2024) as well as cold stored fruits such as banana (*Musa acuminata*), mango (*Mangifera indica*), loquat (*Eriobotrya japonica*), kiwifruit (*Actinidia chinensis*) and peach (Jiao 2021; Hou et al. 2022; Hu et al. 2022; Song et al. 2022; Xu et al. 2023b). Previous investigations have established that both low-temperature conditioning (LTC) and ethylene application effectively mitigate CI in peach fruit, which was accompanied by marked shifts in lipid composition (Wang et al. 2017; Zhu et al. 2019), suggesting that lipid metabolism is critically involved in the development of CI in peach fruit. However, the proteins that regulate lipid remodeling, the specific lipid molecular species involved, and their precise regulatory roles during postharvest cold storage remain undetermined.

While transcriptomic approaches provide invaluable insights, their ability to fully delineate physiological mechanisms is limited in certain circumstances by the spatiotemporal dynamics introduced through post-translational regulations (Vogel and Marcotte 2012; Liu et al. 2016). Proteomics, by contrast, provides a more direct interrogation of functional states by systematically profiling global protein abundance, thereby offering precise insights into dynamic cellular processes and being an important complementary tool to transcriptomic analysis (Bensimon et al. 2012). Consequently, proteomic strategies have become extensively employed in dissecting growth, development, and stress adaptation in a variety of plants, such as tomato (*Solanum lycopersicum*) and oriental melon (*Cucumis melo*) (Guo et al. 2017; Momo et al. 2023; Tan et al. 2025). The application of proteomic approaches has substantially advanced our understanding of fruit physiology under low-temperature storage (Ning et al. 2022; Salazar-Salas et al. 2022; Xu et al. 2023a; Wang et al. 2024). In peach, a 2-D difference gel electrophoresis (DIGE) proteomic strategy was employed to investigate fruit softening and CI, which led to the discovery of a substantial set of novel proteins associated with stress responses and carbohydrate metabolism (Nilo et al. 2010). A proteomic study on chilling-induced mealiness in peach also identified several proteins involved in ethylene synthesis, ABA responses, stress responses, and energy metabolism (Almeida et al. 2016). A recent proteomic study on controlled atmosphere alleviated CI in peach fruit identified several proteins in the energy metabolism pathway (Zhou et al. 2022). However, although proteomic approaches have enabled the identification of an increasing number of proteins associated with the onset of CI, the biological functions of these proteins and their mechanistic contributions to the underlying signaling and response pathways remain largely elusive.

In this study, we performed a proteomic analysis of “Zhonghuashoutao” (“ZHST”) peach fruit subjected to varying storage temperatures and durations and identified a key protein - a Gly-Asp-Ser-Leu (GDSL) domain esterase/lipase (GELP) (commonly classified as EC 3.1.1.-) - PpGELP18, involved in CI. Heterologous stable expression of *PpGELP18* in tobacco (*N. tabacum*) confirmed its role in enhancing chilling tolerance, and subsequent lipid profiling revealed that PpGELP18 elevates key membrane lipids, notably specific unsaturated PLs such as phosphatidylcholines (PC) and phosphatidylethanolamines (PE), thereby improving membrane integrity and fluidity. These findings were further supported by ultrastructural observations of peach fruit, demonstrating that CI leads to a loss of membrane integrity. Collectively, our study identified a previously uncharacterized GELP in peach that contributes to low-temperature adaptation through the remodeling of lipid composition. To our knowledge, the lipid-mediated regulation of plant cold resistance by a GELP has not been previously reported. Thus, these findings provide new insights into the physiological and genetic mechanisms underlying CI in peach and other cold-sensitive species.

## Results

### Phenotype analysis of postharvest CI in peach fruit under different temperatures

CI assessment of “ZHST” peach fruit revealed a clear temperature-dependent response. No visible symptoms were observed in fruit stored at either 0 or 16 °C throughout the storage period. Fruit maintained at 16 °C developed noticeable flesh reddening (Fig. 1a), a phenomenon consistent with our previous findings that temperature regulates anthocyanin accumulation in peach flesh (Zhu et al. 2020; Huang et al. 2024). By contrast, peach fruit stored at intermediate temperatures (5, 8, and 12 °C) exhibited severe IB after 30 d of storage (Fig. 1a). The IB index was substantially elevated in peach fruit stored for 30 and 40 d at 5, 8, and 12 °C. Notably, fruit stored at 5 and 8 °C for 40 d exhibited markedly higher IB values than those at other temperatures (Fig. 1b).

**Figure 1:**
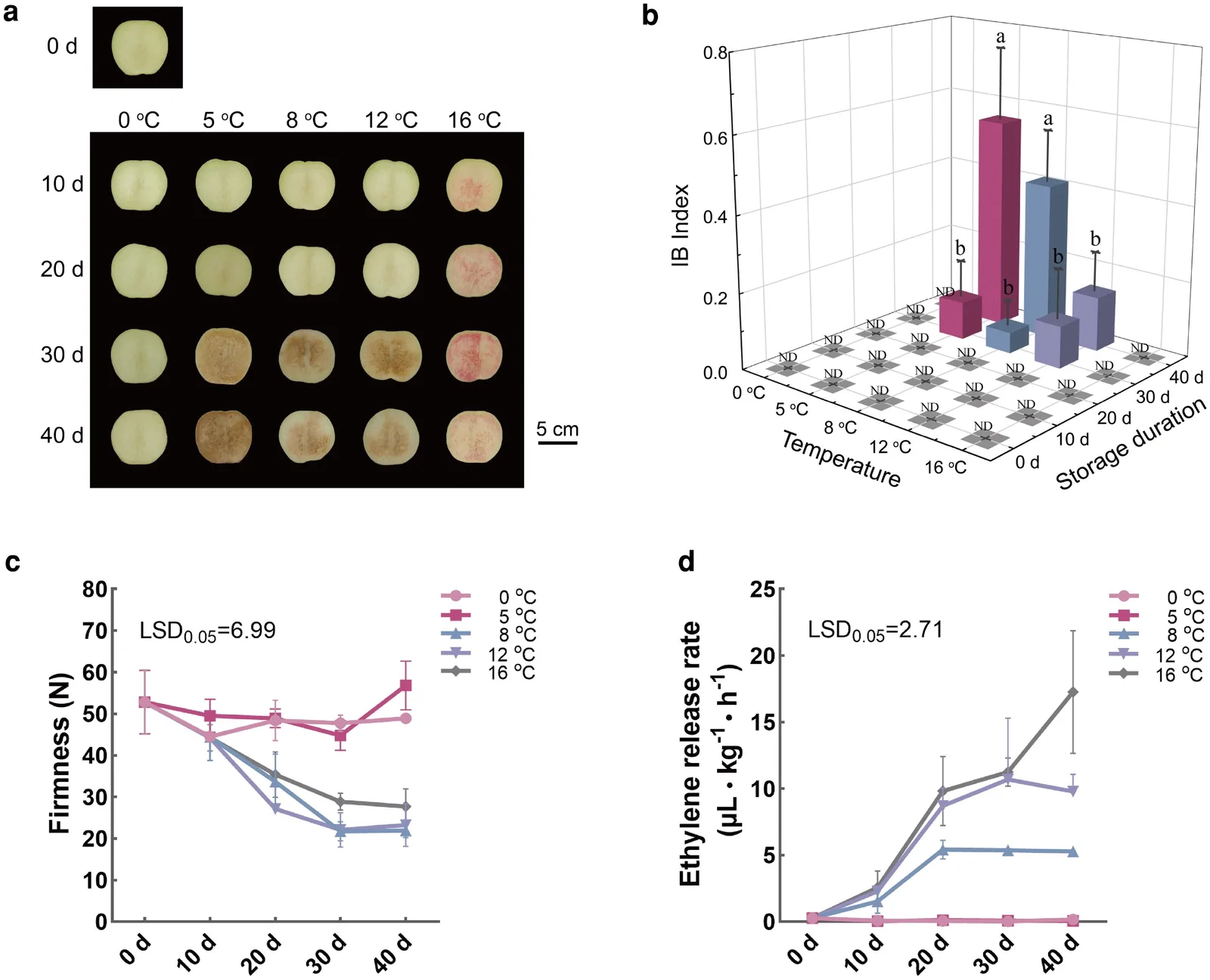
Phenotype and physiological changes in peach fruit during storage at different temperatures. (a) Photographs of fruit flesh. White bar, 5 cm. (b) Internal browning (IB) index. (c) Fruit firmness. (d) Ethylene production rate. Mean ± SE from 3 independent biological replicates were presented. LSD, the least-significant difference at *P* = 0.05. Different lowercase letters denoted a significant difference (*P* < 0.05).

To determine the physiological changes associated with the development of CI-induced IB, fruit firmness and ethylene production rates of peach fruit stored at different temperatures were assessed. Peach fruit stored at 8, 12, and 16 °C exhibited a marked decline in firmness as compared with the fruit stored at 0 and 5 °C throughout the storage period (Fig. 1c). Concurrently, ethylene production rates remained extremely low at 0 and 5 °C during the entire storage duration. However, a substantial increase in ethylene production was observed at 8, 12, and 16 °C during the first 20 d, with the production rate positively correlated with storage temperature (Fig. 1d).

### Identification of key proteins associated with temperature-dependent CI

To elucidate the biochemical mechanisms underlying temperature-dependent CI, we employed a 2-dimensional proteomic strategy. Fruit samples were selected based on 2 criteria: (i) fruit from all storage temperatures collected at 40 d, when symptoms of IB were most severe in some treatments, and (ii) fruit stored at 5 °C, a temperature known to induce CI in peach, at multiple time points to assess temporal changes in protein abundance. To quantify relative protein abundance, tandem mass tag (TMT)-based proteomic analysis across multiple experimental groups (Fig. 2) was conducted, with 3 biological replicates per group. In the proteomic analysis of peach fruit at different temperatures for 40 d, a total of 4,288 proteins were identified at a false discovery rate (FDR) value of 1% (Table S1). After applying a filter to include only proteins with quantification values in all 15 samples, 3,966 proteins were retained for subsequent statistical analyses (Table S2). In the proteomic analysis of peach fruit stored at 5 °C for different days, a total of 4,375 proteins were identified, and 4,300 proteins were quantified across all 15 samples (Tables S3 to S4).

**Figure 2:**
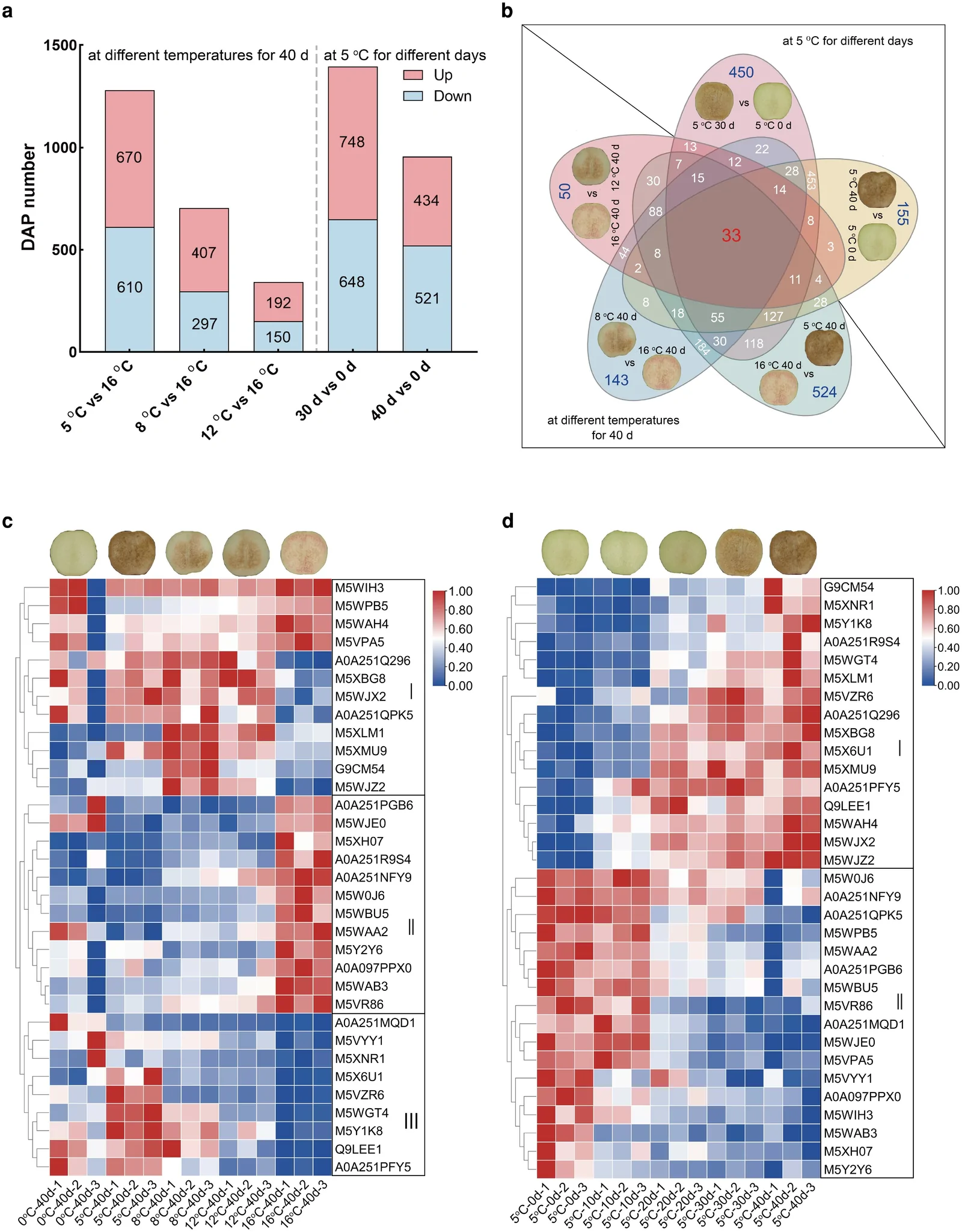
Comprehensive analysis of differentially accumulated proteins (DAPs). (a) Up- or down-regulated proteins between chilling-injured fruit and non-chilling-injured fruit. (b) Venn diagram depicting the overlap of DAPs associated with internal browning. (c) Abundance heatmaps of 33 DAPs from fruit stored at different temperatures for 40 d. (d) Abundance heatmaps of 33 DAPs from fruit stored at 5 °C for different days. Proteins exhibiting a fold change of > 1.2 and a *P* < 0.05 were defined as DAPs. The rows in the heatmap represent proteins, and the columns indicate samples. Each sampling time of peach fruit for analysis has 3 biological replicates, with 3 fruits in each replicate. Red and blue colors indicate high and low expression levels, respectively.

To identify IB-associated proteins, we compared the protein abundance between CI and non-CI fruit with a fold change >1.2 and *P* < 0.05 as the criteria for statistical significance and defined these as differentially accumulated proteins (DAPs). For the temperature effect, fruit from 3 CI temperatures (5, 8, and 12 °C) were compared against those at the non-CI temperature 16 °C (Fig. 2a; Tables S5 to S7). For the temporal effect, fruit at the CI time points (30 and 40 d) at 5 °C were compared against the pre-storage control (0 d) (Fig. 2a; Tables S8 to S9). In the analysis of DAPs at different temperature conditions, comparison between 5 and 16 °C revealed 670 up-regulated and 610 down-regulated DAPs. Similarly, 407 up-regulated and 297 down-regulated DAPs were identified when comparing 8 with 16 °C, while 192 up-regulated and 150 down-regulated DAPs were detected in the 12 versus 16 °C comparison (Fig. 2a). Regarding DAPs analysis over storage duration at 5 °C, 748 up-regulated and 648 down-regulated DAPs were identified at 30 d compared with 0 d, whereas 434 up-regulated and 521 down-regulated DAPs were observed at 40 d relative to 0 d (Fig. 2a). Kyoto Encyclopedia of Genes and Genomes (KEGG) enrichment analysis of DAPs revealed that, under both temperature regimes and storage durations, the top 20 pathways were commonly enriched in “ribosome” “protein processing in endoplasmic reticulum” and “photosynthesis”, indicating that chilling stress broadly disrupts protein synthesis and processing. Notably, the enrichment of the “photosynthesis” pathway in non-photosynthetic peach fruit implied potential chilling-induced damage to plastid structures, whereas the presence of “oxidative phosphorylation” among the top pathways in storage durations suggested additional impairment of mitochondrial function (Figures S1 and S2).

The intersection of these 2 independent analyses identified 33 common DAPs (Fig. 2b; Tables S10 to S11), which represent strong candidates for involvement in IB occurrence. The 33 DAPs were classified into 3 distinct clusters according to their abundance profiles after 40 d at different temperatures (Fig. 2c). Cluster I exhibited a divergent abundance pattern: approximately 67% of its DAPs were markedly reduced at 16 °C, whereas the remaining 33% retained relatively high abundance across all temperature conditions. In contrast, proteins assigned to Cluster II accumulated to significantly higher levels at 16 °C relative to other temperatures. DAPs in Cluster III displayed elevated abundance under chilling temperatures (0, 5, 8, and 12 °C) as compared with 16 °C (Fig. 2c). This profile paralleled the temperature-dependent incidence of IB, implicating these proteins as potential candidates for further functional analysis. Furthermore, analysis across the storage durations at 5 °C classified the DAPs into 2 clusters with inverse abundance patterns (Fig. 2d). Proteins in Cluster I showed increased abundance at late storage (30 and 40 d), concurrent with the progression of IB. Conversely, proteins in Cluster II exhibited a marked downregulation at the same stages (Fig. 2d). To identify DAPs critically associated with IB under CI, we correlated the abundance of the 33 DAPs with the IB index across temperatures and storage durations. As a result, a protein (PRUPE_1G497700) with UniProt code M5Y1K8 was consistently ranked among the top 5 proteins most strongly correlated with IB in both analyses (Tables S12 to S13). Given this consistent and strong association, this protein was selected for further study to elucidate its role in temperature-dependent CI in peach fruit.

### Characterization of protein PpGELP18 in peach

The protein abundance of M5Y1K8 was significantly upregulated at CI temperatures (5, 8, 12, and 16 °C) and during the later stages of storage (Figure S3), suggesting a relationship with chilling-induced IB. *In silico* analysis via NCBI CD-Search and InterPro classified this protein as a member of the plant Ser–Gly–Asn–His (SGNH) lipase family, specifically a subfamily of GDSL-type esterases/lipases (GELP) (Fig. 3a, Figure S4). Phylogenetic analysis revealed that the protein M5Y1K8 shared the closest relationship with AtGELP33 (Figure S5). However, the protein was given the designation PpGELP18, using nomenclature from a prior study on the Rosaceae GELP gene family (Cao et al. 2018).

**Figure 3:**
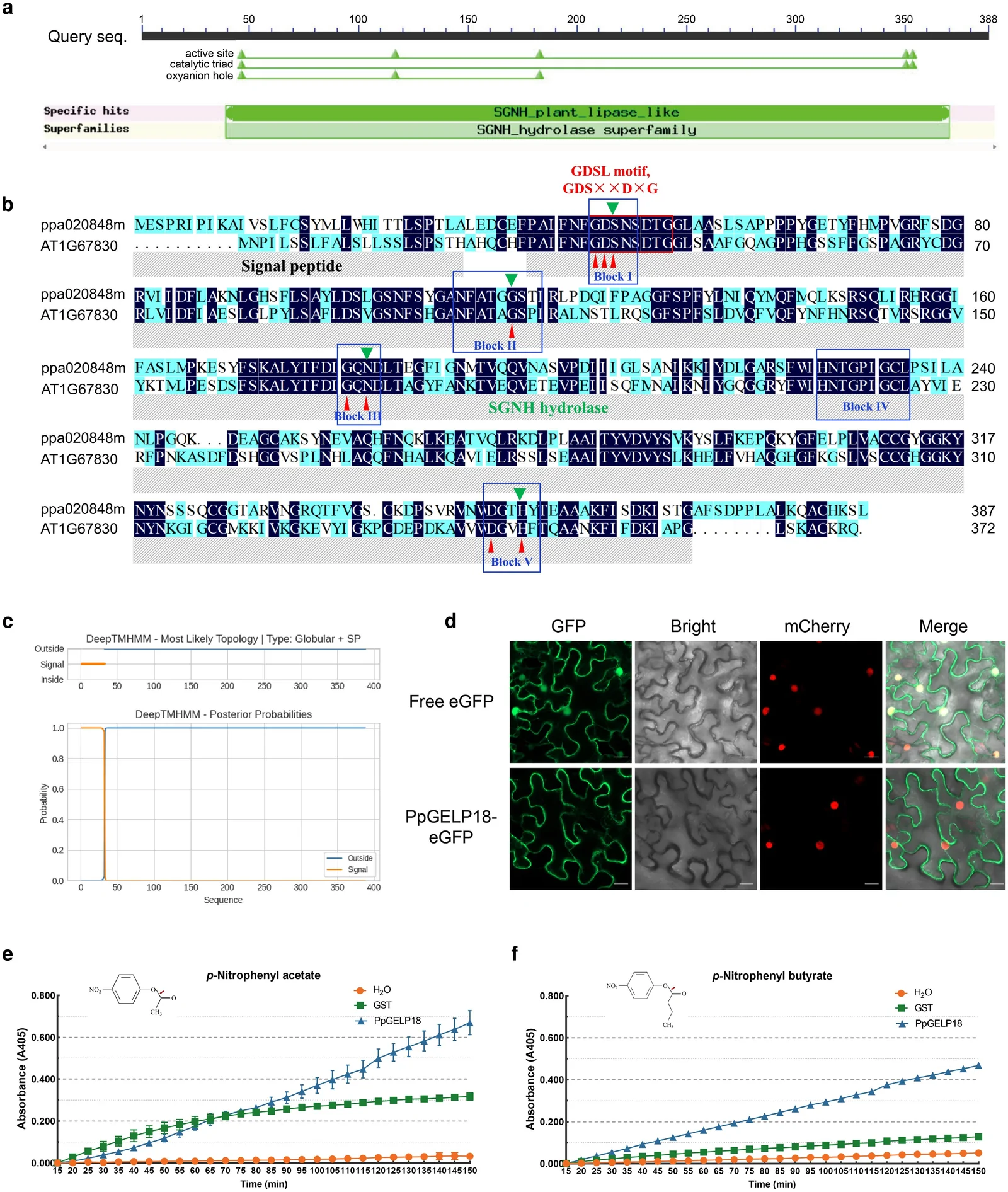
Sequence features, subcellular localization, and enzymatic function analysis of PpGELP18. (a) Predicted conserved domain of PpGELP18. (b) Multiple alignment of protein sequences of PpGELP18 and Arabidopsis AtGELP33. Rectangles: consensus blocks I, II, III, IV and V, as well as GDSL motif; upward and red-filled triangles: absolutely conserved residues; downward and green-filled triangles: invariant catalytic residues. (c) Predicted transmembrane domains of PpGELP18. (d) Subcellular localization of PpGELP18 in mCherry-NLS transgenic *Nicotiana benthamiana* leaves. Bar = 20 μm. E and (f) Lipase activities of PpGELP18 with *p*-nitrophenyl acetate (e) and *p*-nitrophenyl butyrate (f) as substrates. The substrates were incubated with either glutathione *S*-transferase (GST) protein or no protein (H_2_O) as controls. Data are shown as means ± SE (n = 3). Note that the SE bars for certain data points are negligible in size and thus invisible.

A multiple sequence alignment between PpGELP18 and Arabidopsis homolog AtGELP33 was subsequently conducted. The alignment revealed the presence of a canonical serine-containing GDSL motif GDSxxDxG near the N-terminus and 4 characteristic conserved sequence blocks (I-V), which are present in most GELPs. Critically, blocks I, II, III, V contained 1 invariant catalytic residue, Ser, Gly, Asn, and His, respectively (Fig. 3b), which are essential for SGNH hydrolase activity (Upton and Buckley 1995; Akoh et al. 2004; Ding et al. 2019b).

To determine the subcellular localization of PpGELP18, we performed a transmembrane domain prediction using DeepTMHMM 1.0. The analysis predicted an N-terminal 32-amino acid signal peptide (Fig. 3c), suggesting that the mature protein (residues 33-387) was likely extracellular or membrane-associated. To experimentally validate this prediction, we transiently expressed 35S::*PpGELP18* fused to green fluorescent protein gene (*PpGELP18-eGFP*) in leaves of transgenic *N. benthamiana* already stably overexpressing *mCherry* fused to a nuclear localization signal (mCherry-NLS). Confocal microscopy revealed that the GFP signal was predominantly localized to the plasma membrane (Fig. 3d).

The hydrolytic activity of PpGELP18 was verified in vitro where purified PpGELP18 (Figure S6) was incubated with *p-*nitrophenyl acetate and *p-*nitrophenyl butyrate, 2 artificial substrates widely employed for lipase activity assays. Compared with glutathione *S*-transferase (GST) protein and the water control, GST-PpGELP18 exhibited pronounced lipase activity (Fig. 3e and f). Taken together, the membrane localization and hydrolytic activity of PpGELP18 strongly suggests its potential role in membrane-associated processes.

### PpGELP18 enhances the chilling resistance of transgenic tobacco (*N. tabacum*)

To investigate the potential role of *PpGELP18* in the responses to low-temperature stress, we generated stable transgenic tobacco (*N. tabacum*) lines overexpressing *PpGELP18,* obtaining 4 independent transgenic lines (Fig. 4a). qRT-PCR analysis confirmed that all 4 transgenic lines maintained consistently high transcript levels of *PpGELP18* under chilling conditions (Figure S7). Notably, the transgenic lines displayed a marked enhancement in chilling tolerance relative to wild-type (WT). Under standard growth conditions, both transgenic and WT plants exhibited vigorous growth with no discernible phenotypic differences. Following exposure to 0 °C for 14 d, however, WT plants displayed severe wilting and pronounced leaf yellowing, whereas the transgenic plants were less affected (Fig. 4a). Consistent with these phenotypic observations, both WT and transgenic plants showed high maximum photochemical efficiency of photosystem II (*Fv/Fm*) values under normal conditions, whereas they differed significantly in response to chilling stress, where the transgenic lines maintained a higher *Fv/Fm*, with values retaining 30% to 35% of control (before chilling treatment) levels, as compared with a severe reduction to 23% in WT plants (Fig. 4b).

**Figure 4:**
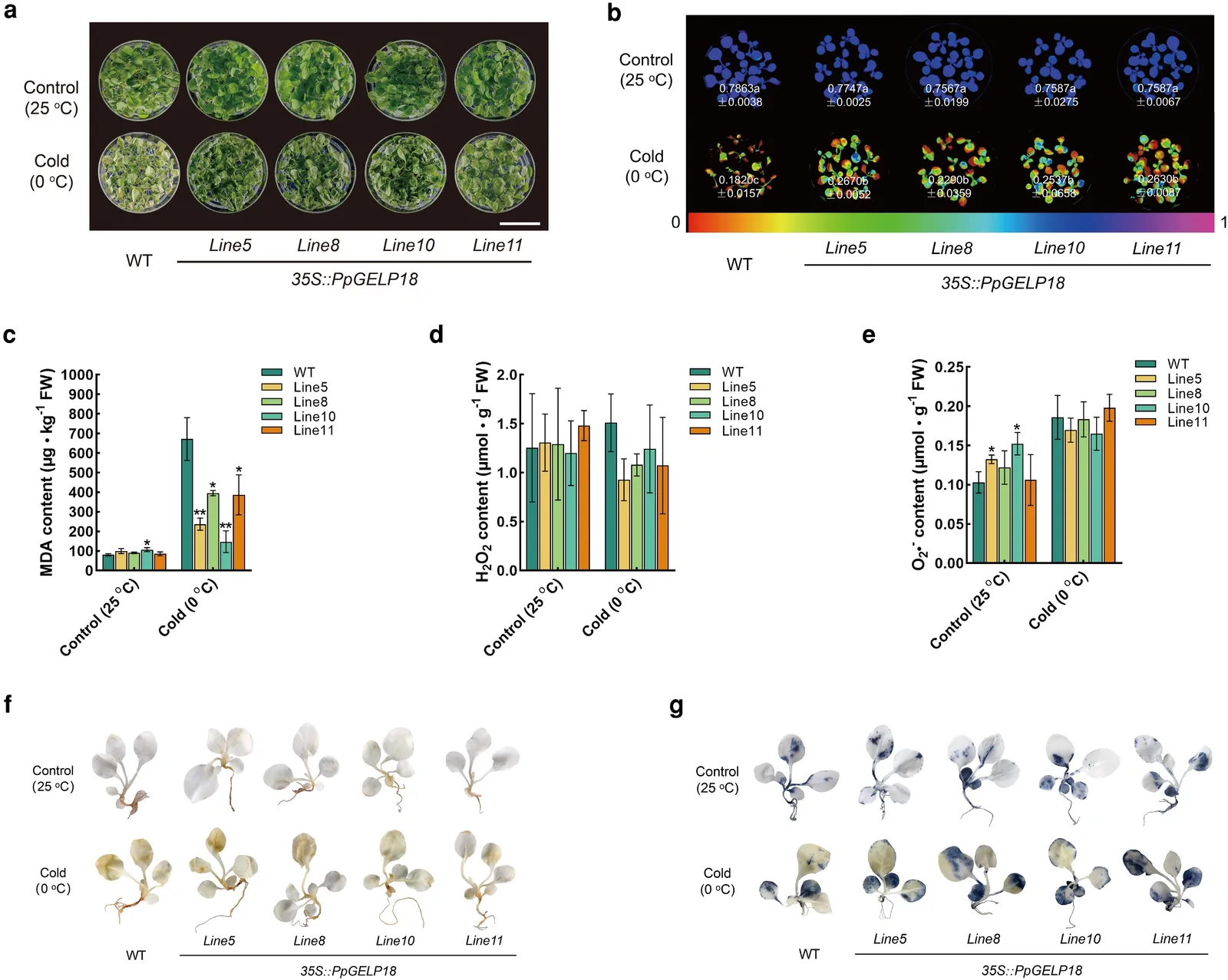
Chilling tolerance assessment of transgenic tobacco (*N. tabacum*) plants overexpressing *PpGELP18* as compared with wild-type (WT). Plants of 21-day-old were subjected to a chilling treatment of 0 °C for 14 d, with plants before chilling treatment serving as the control. (a) Photos. White bar: 5 cm. (b) Maximum photochemical efficiency of photosystem II (*Fv/Fm*). The false color code depicted at the bottom of the images ranges from 0 (black) to 1 (purple). (c) Malondialdehyde (MDA) content. (d) H_2_O_2_ content. (e) O_2_•^−^ content. (f) 3,3′-Diaminobenzidine (DAB) histochemical staining. (g) Nitro blue tetrazolium (NBT) histochemical staining. Different lowercase letters denote a significant difference (*P* < 0.05). Data are presented as means of 3 biological replicates ± SE. Asterisks indicate statistically significant differences compared with the WT under the same condition (* *P* < 0.05; ** *P* < 0.01). For WT or each transgenic line for either control or cold treatment, 9 seedlings were stained and examined, with 1 representative individual seedling shown.

Furthermore, the levels of malondialdehyde (MDA), a marker of membrane lipid peroxidation, increased after chilling treatment, yet the transgenic lines accumulated significantly less MDA, with levels only 22% to 29% of those in the WT (Fig. 4c). To determine whether PpGELP18 enhanced chilling tolerance by regulating intracellular reactive oxygen species (ROS), we measured the levels of H_2_O_2_ and superoxide anion (O_2_^•−^) in WT and transgenic plants before and following chilling treatment. No significant difference was observed between WT and transgenic lines following chilling treatment (Fig. 4d and e). Consistent with these observations, histochemical staining with 3,3′-diaminobenzidine (DAB), for H_2_O_2_, and nitro blue tetrazolium (NBT), for O_2_^•−^, revealed no marked difference between WT and transgenic plants, either before or following chilling treatment (Fig. 4f and g).

### PpGELP18 alters lipid composition of transgenic tobacco leaves under chilling stress

Lipids constitute the fundamental structural components of biomembranes. To investigate the function of PpGELP18 in regulating membrane lipids under low temperature, we conducted a comprehensive lipidomic analysis of tobacco (*N. tabacum*) leaves from plants subjected to chilling treatment. Principal component analysis (PCA) of lipid composition well distinguished the 4 transgenic lines from WT (Figure S8a), with the loading plot identifying the lipid subclasses driving this separation (Figure S8b).

Compared with WT, line 5 showed a 12% reduction in total lipid content, while the other 3 lines exhibited no significant difference (Fig. 5a). To elucidate the potential involvement of PpGELP18 in lipid metabolism, lipids were systematically classified into 6 classes, ie, PLs, lysophospholipids, glycerolipids, glycolipids, sphingolipids, and free fatty acids (FFA), for comparative profiling. The PL subclasses phosphatidylcholine (PC) and phosphatidylethanolamine (PE) accumulated to higher levels in the transgenic lines than in WT, with increments of 24%-55% and 17%-65%, respectively. By contrast, phosphatidic acid (PA) content in the transgenic lines was reduced by 65%-80% (Fig. 5b). Lysophospholipids, hydrolysis products of PLs, were consistently and significantly reduced in all subclasses in transgenic tobacco (*N. tabacum*). Levels of lysophosphatidic acids (LPA), lysophosphatidylcholines (LPC), lysophosphatidylethanolamines (LPE), lysophosphatidylglycerols (LPG), and lysophosphatidylinositols (LPI) all decreased to below half of WT (Fig. 5c). Within the glycerolipid class, monoacylglycerol (MAG) levels showed no significant difference between transgenic lines and WT. However, diacylglycerol (DAG) content decreased by 43%-55%, whereas triacylglycerol (TAG) content increased by 13%-38% relative to WT (Fig. 5d).

**Figure 5:**
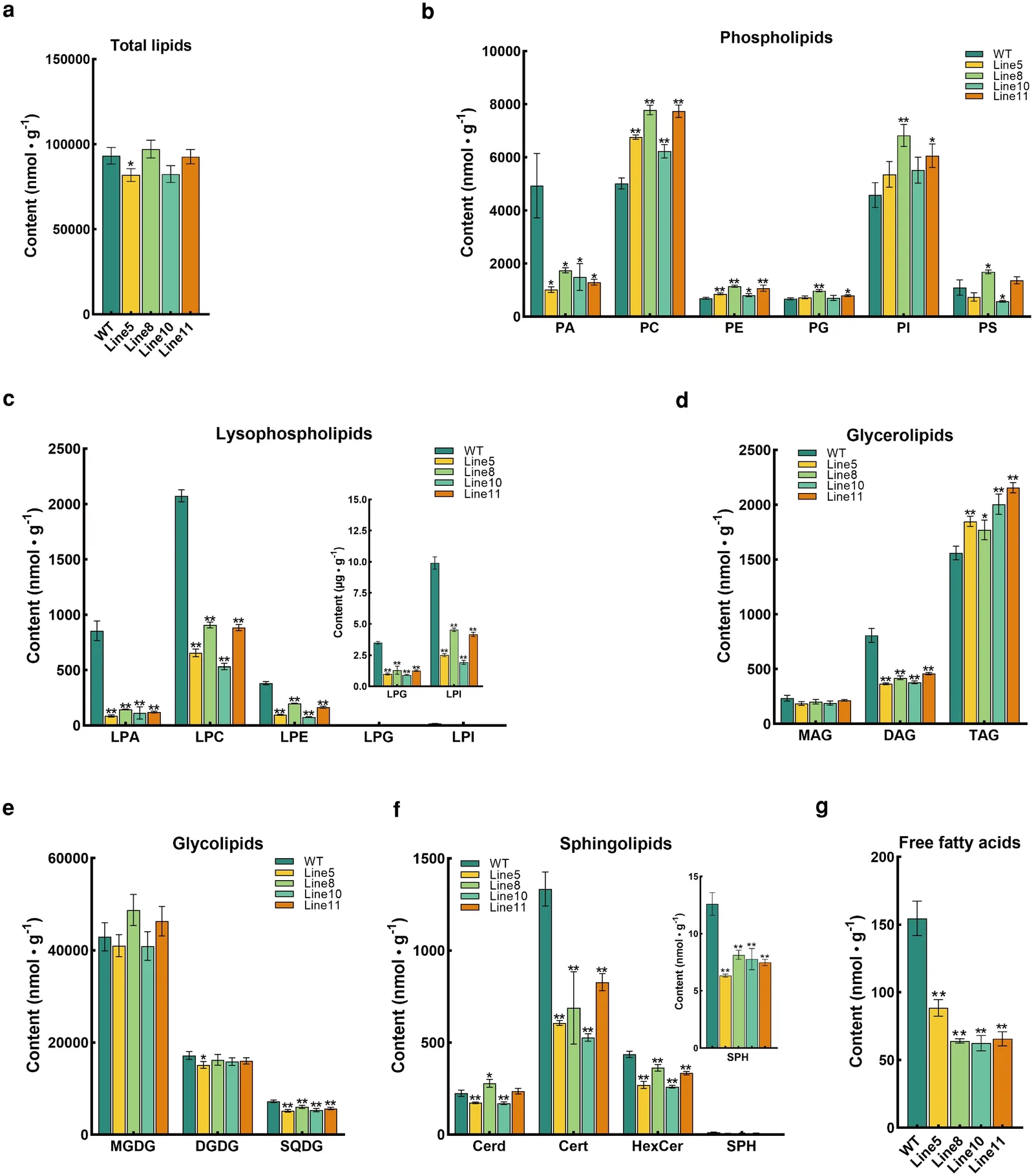
Contents of major lipid subclasses in wild-type (WT) and transgenic tobacco (*N. tabacum*) plants after low-temperature treatment. (a) Total lipids. (b) Phospholipids. (c) Lysophospholipids. (d) Glycerolipids. (e) Glycolipids. (f) Sphingolipids. (g) Free fatty acid. Cerd, dihydroxy ceramide; Cert, trihydroxy ceramide; DAG, diacylglycerol; DGDG, digalactosyldiacylglycerol; HexCer, hexosylceramide; LPA, lysophosphatidic acid; LPC, lysophosphatidylcholine; LPE, lysophosphatidylethanolamine; LPG, lysophosphatidylglycerol; LPI, Lysophosphatidylinositol; MAG, monoacylglycerol; MGDG, monogalactosyldiacylglycerol; PA, phosphatidic acid; PC, phosphatidylcholine; PE, phosphatidylethanolamine; PG, phosphatidylglycerol; PI, phosphatidylinositol; PS, phosphatidylserine; SPH, sphingosine; SQDG, sulfoquinovosyldiacylglycerol; TAG, triacylglycerol.

Among glycolipids, monogalactosyldiacylglycerol (MGDG) and digalactosyldiacylglycerol (DGDG) levels were comparable between transgenic lines and WT, whereas sulfoquinovosyldiacylglycerol (SQDG) level was significantly reduced, by 16%-28%, in the transgenic lines (Fig. 5e). For sphingolipids, the amounts of trihydroxy ceramides (Cert), hexosylceramides (HexCer) and sphingosines (SPH) decreased to 39%-62%, 60%-83% and 50%-65% of WT levels, respectively (Fig. 5f). In addition, FFA content was reduced to 40%-57% of WT levels in the transgenic lines (Fig. 5g). Collectively, significant alterations were observed between transgenic lines and WT in multiple lipid classes, including PLs (PA, PC and PE), all lysophospholipid subclasses, glycerolipids (DAG and TAG), glycolipids (SQDG), and sphingolipids (Cert, HexCer and SPH). This indicates that *PpGELP18* overexpression facilitates the remodeling of diverse lipid subclasses in tobacco (*N. tabacum*) exposed to chilling conditions.

Given that each lipid subclass comprises multiple lipid species, we further analyzed changes in the abundance of individual lipid species in leaves from plants subjected to chilling treatment. The majority of PL species exhibited pronounced enhanced accumulation in *PpGELP18* overexpression lines compared with WT (Fig. 6; Figure S9a). This tendency was also observed in a select subset of glycolipids, including MGDG(14:0_18:3), MGDG(18:1_18:3), MGDG(18:3_18:4), and DGDG(17:0_18:3), as well as in a few TAG species (Fig. 6, Figures S9 and S10). Conversely, the accumulation of the vast majority of lysophospholipid species, half or more of glycerolipid MAG and DAG species, a range of sphingolipid species, and over half of FFA species was significantly reduced (Fig. 6, Figure S9). Notably, among PLs, particularly membrane ones, 16% to 31% of PC, PE, and phosphatidylglycerol (PG) species showed significantly increased amounts in the transgenic lines, whereas approximately half of PA species exhibited decreased accumulation (Fig. 6; Figure S9a).

**Figure 6:**
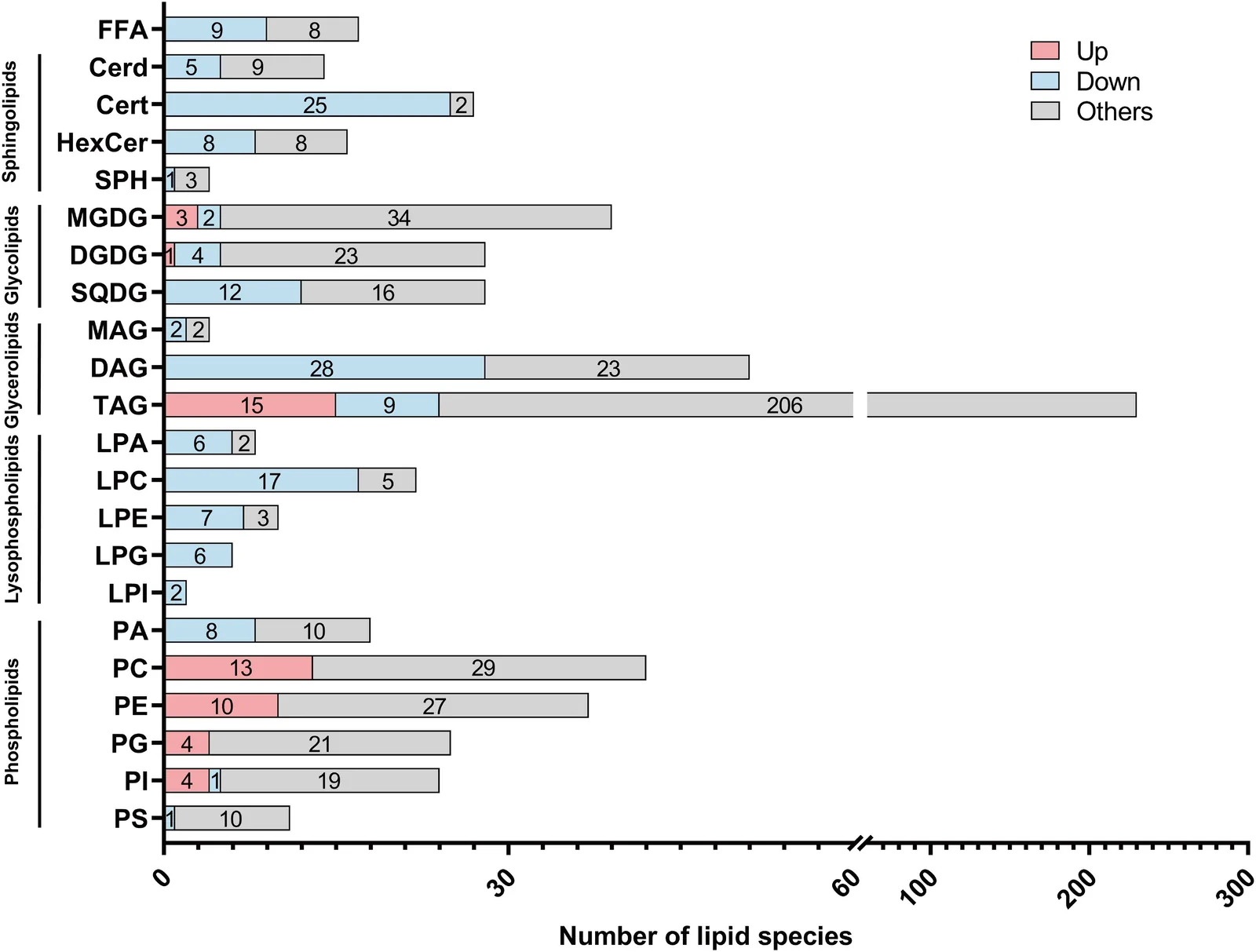
Numbers of differentially accumulated lipid species within each lipid subclass in transgenic versus wild-type (WT) tobacco (*N. tabacum*) plants under chilling stress. Up, number of species with significantly increased abundance in all 4 transgenic lines relative to WT; Down, number of species with significantly decreased abundance in all 4 transgenic lines relative to WT; Others, number of species exhibiting no consistent change across the 4 transgenic lines compared with WT. FFA, free fatty acid. The other abbreviations for lipid compounds are the same as listed in Fig. 5.

### Lipid remodeling occurs during temperature-dependent CI in peach

The lipidomic analysis was extended to peach fruit from different storage temperatures and durations. Peach fruit stored at 5 °C, the temperature with most severe IB, exhibited lower content of total lipids (Fig. 7a) as well as PL subclasses PA, PC, PE and PS, as compared with those at non-CI temperatures (16 °C) (Fig. 7b). Lysophospholipids subclasses LPA, LPC and LPE showed a similar trend, with lower levels at CI temperature 5 °C as compared with non-CI temperature 16 °C (Fig. 7c). Over different storage durations, contents of total lipids as well as PLs overall decreased during storage, and the levels of 2 lysophospholipid subclasses, LPC and LPE, increased (Fig. 7h–j). Within the glycerolipid subclasses, the amounts of DAG and TAG were significantly elevated under CI temperatures, especially at 5 and 8 °C, and also increased notably following 30 d and 40 d of storage at 5 °C (Fig. 7d and k). Among glycolipid subclasses, fruit exhibiting CI-induced IB showed a significantly lower content of MGDG while having a higher content of DGDG (Fig. 7e and l). The content of the sphingolipid subclasses, especially Cert, was lower at 0 and 5 °C (Fig. 7f), yet higher after 30 and 40 d of storage at 5 °C (Fig. 7m). Cerd content was lower in CI-fruit. No significant difference in HexCer or SPH levels were observed across temperatures, however, HexCer content decreased at 30 and 40 days of storage (Fig. 7f and m). No uniform difference was observed in the content of FFA among different storage temperatures and different durations (Fig. 7g and n).

**Figure 7:**
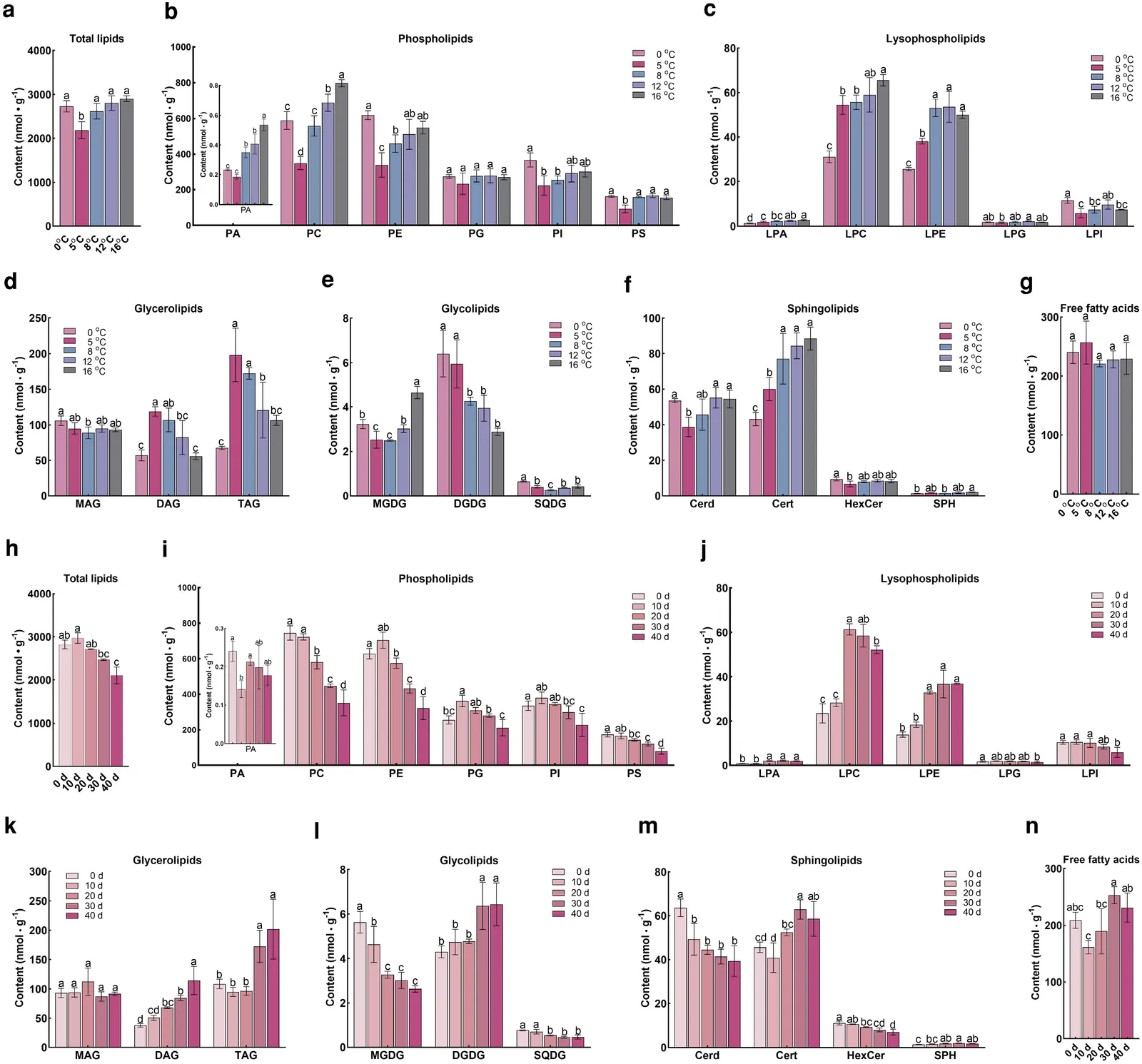
Changes in lipid composition of peach fruit during storage. (a to g) Fruit stored at different temperatures for 40 d. (h to n) Fruit stored at 5 °C for different durations; (a and h) Total lipids. (b and i) Phospholipids. (c and j) Lysophospholipids. (d and k) Glycerolipids. (e and l) Glycolipids. (f and m) Sphingolipids. (g and n) Free fatty acids. Mean ± SE from 3 independent biological replicates were presented. Different lowercase letters denoted a significant difference (*P* < 0.05). The abbreviations for lipid compounds are the same as listed in Fig. 5.

More importantly, the protein abundance of PpGELP18 was significantly higher in fruit stored at CI temperatures (0, 5, 8, and 12 °C) as compared with the non-CI temperature of 16 °C (Figure S3a). Furthermore, its level increased progressively with storage duration, showing the highest accumulation at 40 d (Figure S3b). Collectively, our results from transgenic tobacco (*N. tabacum*) and peach fruit revealed a consistent lipid remodeling response associated with PpGELP18-mediated chilling tolerance, manifesting as elevated PL levels and reductions in lysophospholipids and DAGs.

### CI in peach fruit is accompanied by tonoplast and plasma membrane damage

Based on the observed changes in membrane structural components (eg, PLs and glycolipids) in peach fruit and tobacco (*N. tabacum*) leaves and the plasma membrane localization of PpGELP18, we aimed to determine whether these changes affected membrane structure and integrity during the development of CI in peach fruit. Ultrastructural analysis revealed severe disruption of the tonoplast and plasma membrane, which appeared discontinuous and fragmented, in peach fruit stored at 0 and 5 °C (Fig. 8). Fruit stored at 8 and 12 °C exhibited tonoplast rupture and plasma membrane detachment from the cell wall. Conversely, fruit stored at 16 °C maintained the integrity of both the tonoplast and plasma membrane, which enclosed a homogeneous layer of cytoplasm (Fig. 8). Ultrastructural observation of peach fruit stored at 5 °C revealed a progressive ultrastructural disruption. The plasma membrane and tonoplast were clear, intact, and tightly appressed to the cell wall at 0 d. By day 10, small vesicles began to appear in the cytoplasm, although the tonoplast and plasma membrane remained largely intact. The number of cytoplasmic vesicles increased substantially by day 20 and 30. Ultimately, by day 40, severe disruption of both the plasma membrane and tonoplast became evident (Fig. 8).

**Figure 8:**
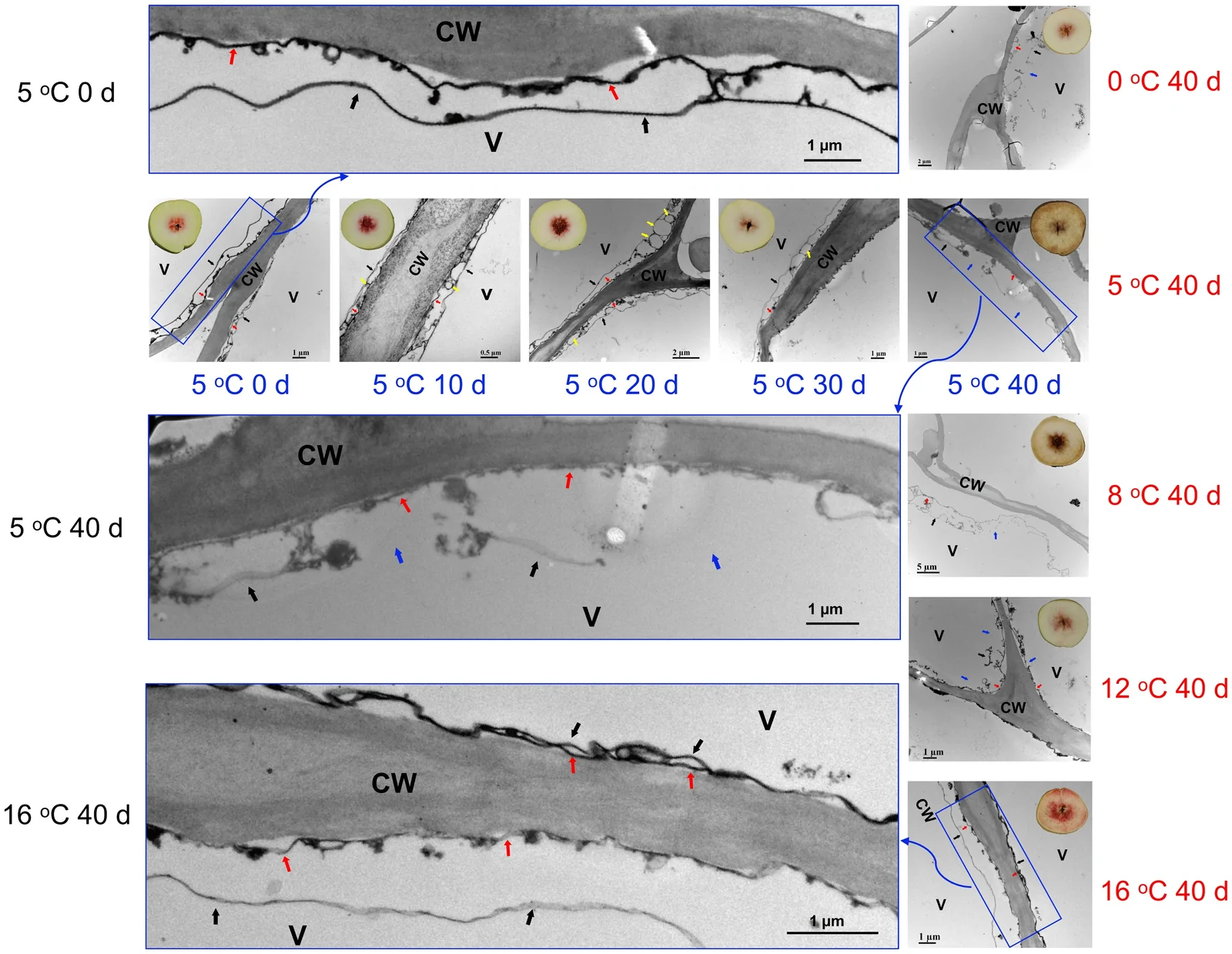
Ultrastructure of peach fruit at different temperatures and storage durations. CW, cell wall; V, vacuole. Black arrows indicate the tonoplast; Red arrows indicate the plasma membrane; Yellow arrows indicate vesicles; Blue arrows point to sites of membrane damage. Note that the scale bars vary to show detail and context.

We also investigated the effects of *PpGELP18* overexpression on ultrastructural changes in tobacco leaves. Before chilling treatment, WT and transgenic plants showed similar ultrastructure, with an intact central vacuole that confines the chloroplasts and mitochondria to the periphery between the plasma membrane and the tonoplast. After chilling treatment, however, WT plants exhibited severe membrane damage, vacuolar rupture, disorganized cellular contents, and swollen, distorted chloroplasts. By contrast, transgenic lines maintained peripheral organelle localization (Figure S11).

These results indicated that the alterations in membrane lipid composition during CI, particularly the reduction in structural components such as PLs and glycolipids, compromised the structure and integrity of membranes. PpGELP18 may function in defense against CI in peach fruit by promoting lipid remodeling that elevates the content of structural membrane components, ultimately preserving membrane integrity.

## Discussion

### GELP mediates abiotic stress tolerance in plants

GELPs were initially characterized during investigations into lipases from *Aeromonas hydrophila* (Upton and Buckley 1995), and subsequently reported to function in diverse biological processes, such as hormone signaling (Kwon et al. 2009; Zhao et al. 2020), seed germination (Ding et al. 2024a), cuticle formation (Zhai et al. 2025), and suberin biosynthesis (Ursache et al. 2021; Zhang et al. 2022). Genomic technologies have facilitated the genome-wide identification of *GELP* gene families across diverse plant species, and the expression profiles of these genes under various abiotic stresses have been characterized. In apple (*Malus domestica*), the promoters of *GELP* genes are enriched with cis-elements associated with abiotic stress responses, and the expression of multiple members was found to be significantly altered by cold stress (Yuan et al. 2025). In *Ricinus communis,* expression of several *RcGELPs* responds to heat and salt stresses (de Almeida et al. 2024). Furthermore, heterologous expression of the *GELP* gene *AmGDSL1* from *Agropyron mongolicum* in *N. benthamiana* conferred significantly enhanced drought tolerance, as evidenced by reduced leaf wilting and improved root architecture in transgenic plants (Yan et al. 2024).

GELPs have also been implicated in the response to low-temperature stress. In *Arabidopsis*, overexpression of *AtCBF1* and *AtCBF2* resulted in the pronounced induction of a *GELP* member (Locus ID *At2g24560*) under low temperature (Gilmour, et al. 2004). Similarly, improved cold tolerance in rice overexpressing the stress-responsive NAC transcription factor gene *SNAC2* was associated with the upregulation of a gene encoding a GDSL-like lipase/acylhydrolase (Hu et al. 2008). Recently, it was reported that overexpression of *CpGLIP1*, a *GELP* from *Chimonanthus praecox*, in both Arabidopsis and poplar conferred increased resistance to drought and freezing (Liu et al. 2024). However, direct evidence elucidating the precise functions of GELPs in plant responses to low-temperature stress is lacking. In the present study, comparative proteomic analysis of peach fruit stored under different temperatures identified a key protein, PpGELP18, associated with CI occurrence. Heterologous stable expression of *PpGELP18* in tobacco (*N. tabacum*) significantly enhanced chilling tolerance (Fig. 4). Furthermore, lipidomic profiling revealed ongoing lipid remodeling during peach CI, a process concomitant with a significant increase in PpGELP18 protein abundance under chilling stress (Fig. 7, Figure S3).

Previous research has implicated that GELP raises abiotic stress tolerance by potentially maintaining membrane homeostasis and osmotic adjustment. For instance, under drought stress, Arabidopsis and poplar (*Populus tremula* × *P. alba*) overexpressing *CpGLIP1* exhibited reduced electrolyte leakage and water loss, indicating enhanced membrane integrity. Furthermore, under chilling stress, transgenic plants overexpressing *CpGLIP1* accumulated higher levels of proline, thereby contributing to osmotic adjustment and improved cellular protection (Liu et al. 2024). Consistent with this, we observed a significant decrease in MDA content in *PpGELP18*-overexpressing transgenic tobacco (*N. tabacum*) plants under chilling stress (Fig. 4c), indicating mitigated oxidative damage to the plasma membrane. Meanwhile, some other research has implicated that GELP enhances abiotic stress tolerance, potentially through the modulation of cellular redox homeostasis. It has been demonstrated that the GELP protein AmGDSL1 from *Agropyron mongolicum* conferred drought tolerance by enhancing the activity of ROS-scavenging enzymes to alleviate oxidative damage under drought stress (Yan et al. 2024). However, research in rice revealed that OsHTA1, a GELP member, negatively regulated thermotolerance by modulating the accumulation of ROS (Su et al. 2024). In this study, no significant alteration in H_2_O_2_ or ROS levels was detected in transgenic tobacco (*N. tabacum*) following chilling treatment (Fig. 4d and e).

### GELP is a key player in plant lipid metabolism

In this current study, subcellular localization analysis revealed that PpGELP18 was a plasma membrane-localized protein (Fig. 3d). Biochemical assay demonstrated its in vitro esterase activity (Fig. 3e and f). Consistent with its biochemical function, transgenic tobacco (*N. tabacum*) plants overexpressing *PpGELP18* showed significant alterations in lipid profiles under chilling stress (Figs. 5 and 6, Figures S9 and S10).

GELPs play diverse roles in lipid metabolism. For instance, in lipid remodeling, it was demonstrated that the rice GELP OsMHZ11 functioned as a phospholipase A_2_ (PLA_2_, EC 3.1.1.4), hydrolyzing PC, PE, PS, and PG into lysophospholipids and FFAs. The released fatty acids can then serve as substrates for sterol-acyltransferases to generate phytosteryl esters (Zhao et al. 2020). Furthermore, rice GELP proteins OsGLIP1 and OsGLIP2 potentially hydrolyze specific lipid substrates, such as TAG and PA, to modulate the accumulation of downstream MGDG and DGDG (Gao et al. 2017). In *Brassica napus*, the GELP protein BnGDSL1 was positively correlated with fatty acid β-oxidation, and silencing *BnGDSL1* altered fatty acid composition, increasing the level of oleic acid (C18:1) while decreasing the levels of linoleic (C18:2) and linolenic (C18:3) acids (Ding et al. 2019a), and another GELP named BnaC07.GLIP1 could promote the accumulation of specific PLs, including PE, PC, and PA (Ding et al. 2024a). In this study, we proposed that PpGELP18 promotes the accumulation of membrane-structural lipids, especially unsaturated PLs (Fig. 5, Figure S9). This process may be driven by the hydrolytic activity of PpGELP18 on alternative lipid substrates, releasing free fatty acids that could, in turn, serve as precursors for the synthesis of structural lipids.

GELPs are also reported to be involved in lipid signal transduction. A vascular-enriched, stress-inducible GELP from *Arabidopsis* bound lipids and was proposed to mediate the transport of PA and other signaling molecules, thereby facilitating intercellular and long-distance communication (Barbaglia et al. 2016). In this study, peach fruit PA content increased with elevated storage temperatures (Fig. 7b), whereas no significant changes were observed across different storage durations at 5 °C (Fig. 7i), implying that PA might act as a signaling molecule participating in response to temperature variations. In contrast, PA level was significantly reduced in transgenic tobacco (*N. tabacum*) (Fig. 5b, Figure S9a), indicating that the improved chilling tolerance conferred by PpGELP18 is unlikely to be mediated through PA accumulation. Whether this PA reduction directly contributes to chilling tolerance, or merely accompanies PpGELP18-mediated lipid remodeling, remains unclear.

### Ultrastructural alterations in plant cells during chilling stress

During chilling stress, the disruption of cellular ultrastructure constitutes a pivotal event contributing to physiological dysfunction in plants. These ultrastructural changes include reduced grana stacking in chloroplasts (Mawson and Cummins 1991), underdeveloped cristae, obscured internal membranes, and a reduction in mitochondrion volume (Yin et al. 2009), as well as vacuolar homogeneity disruption, tonoplast rupture, and plasma membrane disintegration (Ishikawa 1996). Chilling stress induces chloroplast swelling and thylakoid dilation in cucumber (*Cucumis sativus*) leaves (Wise and Naylor 1987), and similar thylakoid and envelope disruptions occur in alfalfa (*Medicago sativa*) under chilling stress (Liu et al. 2025). Comparable ultrastructural abnormalities have also been observed in leaves of WT plants after chilling treatment (Figure S11). The ultrastructural disruption occurred in the plasma membrane, and the tonoplast has also been observed to be disrupted in chilling-sensitive fruits during cold storage. In healthy peach fruit, the cells exhibit a plasma membrane tightly appressed to the cell wall, an intact tonoplast, and a thin cytoplasmic layer. CI causes the plasma membrane to become irregular and detach from the cell wall. Under severe CI, the cellular structure degraded completely, leading to total plasmolysis and tonoplast rupture (Luza et al. 1992). Consistent with these findings, we observed severe disruption of both the plasma membrane and tonoplast in CI-fruit (Fig. 8), whereas vacuoles were more intact in chilling-treated *PpGELP18* overexpressing tobacco (*N. tabacum*) plants (Figure S11). In addition, apparent detachment of the plasma membrane from the cell wall was frequently observed (Fig. 8). During the progression of CI, a marked increase in vesicles was detected between the vacuoles and the plasma membrane as storage duration increased (Fig. 8). Previously in other plants, vesicles were proposed to have a potential role in cellular defense by sequestering damaged lipids or transporting antioxidative compounds, though the exact mechanisms remain unclear (Wise et al. 1983; Musser et al. 1984; Wise and Naylor 1987). In addition to the loss of vacuolar homogeneity and the accumulation of dense material at the tonoplast under chilling stress, tonoplast rupture and plasma membrane disintegration have been regarded as central events in cellular degradation in suspension-cultured callus cells derived from mung bean (*Vigna radiata*) cotyledons (Ishikawa 1996). In peach mesocarp cells, where the vacuole occupies most of the cell volume, tonoplast integrity is essential to prevent storage disorders. Vacuolar disruption may activate CI-related enzymatic processes that lead to IB. Although peaches stored at 0, 5, 8, and 12 °C for 40 d all exhibited varying degrees of tonoplast damage (Fig. 8), IB was not observed in fruit kept at 0 °C until they were transferred to the shelf (20 °C) (Figure S12). This phenomenon could be attributed to the suppressed enzymatic activity at 0 °C.

### Lipid metabolism plays a critical role in plant cold tolerance

In this study, we aimed to elucidate the mechanism of ultrastructural damage under chilling stress by investigating alterations in lipids. PLs serve as fundamental structural components of biomembranes, and the degree of unsaturation in their fatty acyl chains plays a critical role in modulating membrane fluidity (Ernst et al. 2016). In our previous investigation, we observed a marked increase in the levels of specific unsaturated PL species, PC(16:1_20:5), PC(18:4_20:5), and PC(18:3_18:3), in LTC-treated fruit. These lipid species were present at concentrations approximately fourfold higher than those detected in control fruit (Wang et al. 2017).

The molecular species of PLs critically modulated membrane curvature (Harayama and Riezman 2018). Cylindrical PLs, including PC and PS, facilitated the formation of flat membranes. In contrast, conical PLs such as PE and PA promoted membrane invagination and negative curvature. Conversely, inverted-conical lipids, lysophospholipids, and PI induced membrane protrusion and positive curvature (Harayama and Riezman 2018). In peach fruit, the contents of PC, PE, and PS were significantly lower at 5 °C than at other temperatures (Fig. 7b), and a pronounced reduction in these compounds was observed during 40-day storage at 5 °C (Fig. 7i). In contrast, *PpGELP18* overexpressing tobacco (*N. tabacum*) exhibited elevated levels of PC and PE, along with decreased contents of all lysophospholipid subclasses (Fig. 5b and c). In addition, it was found that cold stress led to DAG accumulation at the plasma membrane in Arabidopsis rosette leaves, which was accompanied by impaired membrane integrity (Ruiz-Lopez et al. 2021). In this study, DAG levels were elevated in chilling-injured peach fruit (Fig. 7d and k) yet reduced in transgenic tobacco (*N. tabacum*) plants which were more chilling-tolerant (Fig. 5d, Figure S9b). Together, these data suggested that the structural integrity of biomembranes was compromised during CI, a conclusion further supported by ultrastructural observations of peach fruit cells (Fig. 8). Analysis of PL subclasses revealed that most species of PC, PE, PG, and PI were significantly upregulated in transgenic lines (Figure S9a), and these PLs were generally characterized by a high degree of unsaturation. This suggested a shift in membrane lipid composition toward more unsaturated lipids in response to chilling stress.

Based on the observed lipidomic alterations, we propose that PpGELP18 contributes to the maintenance of membrane homeostasis under chilling stress by selectively remodeling PL composition. Specifically, the upregulation of unsaturated PC and PE species, coupled with the downregulation of lysophospholipids and DAG in transgenic tobacco (*N. tabacum*), is likely to enhance membrane fluidity and structural integrity at low temperatures (Fig. 5, Figure S9a). In addition, transgenic tobacco (*N. tabacum*) under chilling stress exhibited reduced levels of SQDG, Cer, HexCer, SPH, and FFA (Fig. 5), whereas peach fruit did not exhibit comparable trends associated with chilling tolerance (Fig. 7). Meanwhile, TAG content was elevated in both the chilling-injured peach fruit and transgenic tobacco (*N. tabacum*) lines, which were more chilling-tolerant (Fig. 5d; Fig. 7d and k). These discrepancies likely reflect species-specific or organ-contrasted differences in lipid remodeling regulation and warrant further investigation.

## Conclusions

In summary, our findings suggest a potential role for PpGELP18, a GDSL esterase/lipase family member, in modulating CI in postharvest peach fruit. Functional characterization through heterologous stable expression in transgenic tobacco (*N. tabacum*) demonstrated that PpGELP18 enhances chilling tolerance, likely through the remodeling of membrane lipid composition. Specifically, PpGELP18 overexpression elevated the abundance of unsaturated PLs, including PC and PE, while lysophospholipids and DAG levels were reduced. These changes were inferred to enhance membrane fluidity and structural integrity under low-temperature conditions. In peach fruit, exposure to low temperatures (5, 8, and 12 °C) induced PL decline and loss of membrane integrity in both the plasma membrane and tonoplast, ultimately leading to metabolic dysfunction and IB. In contrast, at 16 °C, where membranes remain intact and PpGELP18 abundance is low, the reduced demand for maintaining unsaturated PLs under non-chilling conditions is implied.

## Materials and methods

### Plant materials and treatment

Peach (*P. persica* (L.) Batsch cv. Zhonghuashoutao) fruit were harvested at commercial maturity from an orchard in Zibo (Shandong, China). On the day of harvest, fruit were transported to the laboratory and selected for uniformity in size and absence of physical damage or pathological symptoms. The selected fruit were randomly separated into 5 groups and stored at 0, 5, 8, 12, and 16 °C, respectively. Mesocarp was sampled from each temperature treatment at 10-day intervals throughout 40-day storage, with 3 biological replicates and 3 fruit for each replicate, immediately frozen in liquid nitrogen, and stored at −80 °C for subsequent analysis. After 40 d of storage, all remaining fruit were transferred to 20 °C for 3-day shelf life.

### IB, firmness and ethylene analyses

All physiological assessments were performed using the method described in a previous study (Zhu et al. 2021). The IB index was determined by evaluating the browning degree of the flesh and calculated using the following formula: IB index = 100% × Σ[(IB scale) × (number of fruit at that IB scale)]/[5 × total number of fruit evaluated]. Firmness was determined after removing a 1-mm-thick slice of skin from opposite sides at the equator of each fruit by using a texture analyzer (TA-XT2i Plus; Stable Micro Systems Ltd., Surrey, UK) fitted with a 7.5 mm-diameter probe. For the measurement of ethylene production rate, 3 fruits per biological replicate were sealed in a 3.4-L plastic container. After sealing for 1 h, 1 mL gas sample was collected for ethylene production analysis using gas chromatography (Agilent Technologies 7890B GC System, Santa Clara, CA, United States).

### Transmission electron microscopy

Small tissue segments (approximately 1 × 3 × 1 mm^3^) excised from the peach mesocarp and tobacco (*N. tabacum*) leaves were fixed in 2.5% glutaraldehyde overnight and post-fixed in 1% osmium tetroxide, with intermediate phosphate buffer (0.1 M, pH 7.0) washes. After dehydration through a graded ethanol and acetone series, the samples were infiltrated and embedded in Spurr's resin. Ultrathin sections (70–90 nm) were cut with an ultramicrotome (LEICA EM UC7, Leica Microsystems, Wetzlar, Germany), double-stained with uranyl acetate and lead citrate, and examined using a Hitachi H-7650 transmission electron microscope (Hitachi High-Technologies Corporation, Tokyo, Japan).

### Histochemical staining

The histochemical detection of H_2_O_2_ and O_2_^•−^ in plant tissues was performed using DAB and NBT staining (Leagene Co., Ltd., Beijing, China), respectively. Briefly, seedlings were collected, rinsed, and blotted dry. Samples were immersed in freshly prepared DAB (1 mg/mL in phosphate buffer) or NBT staining solutions and incubated in darkness at room temperature for 2–6 h. Following staining, all samples were rinsed with distilled water and subsequently decolorized in 95% ethanol at 40 °C (3–16 h for DAB-stained samples; 12 h for NBT-stained samples) to remove chlorophylls. The decolorized samples were then rinsed again and photographed.

### MDA, H_2_O_2_ and O_2_^•−^ content measurements

The MDA content was determined according to the previous method (Zhu et al. 2021) with slight modifications. Briefly, frozen leaf samples (0.1 g) were homogenized in liquid nitrogen and extracted with 1 mL of 10% trichloroacetic acid (TCA). After centrifugation, the MDA in the supernatant reacted with thiobarbituric acid (TBA) at 100 °C for 20 min. The absorbance of the product was measured at 532 nm, and the MDA content was calculated and expressed as µg•kg^−1^ fresh weight. The H_2_O_2_ content was determined using the Hydrogen Peroxide Content Assay Kit (Solarbio Science and Technology Co., Ltd., Beijing, China) following the manufacturer's protocol with minor modifications. Frozen leaf samples (0.1 g) were homogenized in liquid nitrogen and extracted with 1 mL of pre-chilled acetone on ice. After centrifugation, the H_2_O_2_ content in the supernatant was determined by measuring the absorbance at 415 nm of the yellow complex formed by the reaction with titanium sulfate. The O_2_^•−^ content was quantified with the Superoxide Anion Activity Content Assay Kit (Solarbio Science and Technology Co., Ltd., Beijing, China). Briefly, hydroxylamine hydrochloride was added to convert O_2_^•−^ into nitrite, and then specific reagents were added to convert the nitrite into a colored azo derivative. The absorbance was measured at 530 nm, and the concentration was calculated according to the provided protocol.

### Protein purification and lipase activity assay

The coding sequence of *PpGELP18* was cloned into the pGEX-4T-1 vector. Recombinant plasmids were transformed into *Escherichia coli* Rosetta (DE3) to generate GST fusion proteins. PpGELP18-GST protein was induced by 0.2 mM β-D-1-thiogalactopyranoside (IPTG) at 16 °C for 20 h. Subsequently, the cells underwent sonication, and the fusion proteins were isolated from the supernatants using the GST-tag Protein Purification Kit (Beyotime Biotechnology, Shanghai, China). Lipase activity was assayed according to the described method (Baudouin et al. 1997; Gao et al. 2017). The standard reaction mixture consisted of 0.5 M HEPES (pH 6.5), 1 mM substrate (*p*-nitrophenyl acetate or *p*-nitrophenyl butyrate), and 2 μg of recombinant protein. Reactions were incubated at 30 °C for 150 min, and the absorbance at 405 nm was recorded at 5-min intervals, commencing 15 min after the start of the reaction.

### Quantitative proteomics analysis

Proteome analysis of peach fruit was performed by the staff at Shanghai Applied Protein Technology Co. Ltd. (Shanghai, China). Proteins were extracted with SDT buffer (4% SDS, 100 mM Tris-HCl, 1 mM dithiothreitol (DTT), pH 7.6) and quantified with a bicinchoninic acid (BCA) assay kit (Bio-Rad Laboratories, Hercules, CA, United States). After the extraction, protein samples (20 μg) were separated with 12.5% sodium dodecyl sulfate—polyacrylamide gel electrophoresis (SDS-PAGE) under a constant current of 14 mA for 90 min, and Coomassie Brilliant Blue R-250 staining was used to display the bands. A portion of 200 μg of protein was taken from each sample for protein digestion and processed by the filter-aided sample preparation (FASP) method: first, SDS and DTT were removed, then the protein was alkylated with iodoacetamide, and finally digested with trypsin at 37 °C overnight. The resulting peptides were desalted using C_18_ cartridges (Empore^TM^ SPE Cartridges C_18_ (standard density), bed I.D. 7 mm, volume 3 mL, Sigma-Aldrich, St. Louis, MO, United States), concentrated via vacuum centrifugation, and dissolved in 0.1% formic acid. Peptide concentrations were determined by UV absorbance at 280 nm. For quantitative proteomics, a portion of 100 μg of peptide was extracted from each sample, and they were labeled using TMT reagents according to the manufacturer's protocols (Thermo Fisher Scientific, Waltham, MA, United States). Subsequently, the labeled peptide segments were separated using a Pierce High pH Reversed-Phase Peptide Fractionation Kit (Thermo Fisher Scientific, Waltham, MA, United States), and 10 separation fractions were generated through acetonitrile gradient elution. Then, the samples were desalted and concentrated. LC–MS/MS measurements were performed on a Q Exactive mass spectrometer (Thermo Fisher Scientific, Waltham, MA, United States) coupled to an Easy-nLC system (Proxeon Biosystems, now Thermo Fisher Scientific, Waltham, MA, United States). Peptides were separated on a C_18_ analytical column using a linear acetonitrile gradient. Data were acquired in data-dependent mode, with survey scans at a resolution of 70,000 and Higher-energy C-trap Dissociation (HCD) fragmentation of the top 10 precursor ions. Raw files were processed with MASCOT (Matrix Science, London, UK; version 2.2) embedded in Proteome Discoverer 1.4 for protein identification and quantification.

### Lipidomics analysis

Lipid content in peach fruit and transgenic tobacco (*N*. *tabacum*) leaf was measured on an AB Sciex QTRAP 6500 LC–MS/MS system. The analysis was carried out by MetWare (Wuhan, China). Lipids were extracted with a methanol/methyl tert-butyl ether (MTBE) mixture (1:3, v/v). Each sample was homogenized with a steel bead with an internal diameter of 4 mm. Ultrapure water was then added, and phase separation was allowed to occur at 4 °C. The upper organic phase was collected and dried. The dried residue was dissolved in acetonitrile/isopropanol (1:1, v/v) for LC–MS analysis. Lipid profiling was performed on an ultra-performance liquid chromatography (UPLC) system (ExionLC AD, Sciex, Framingham, MA, United States) that was coupled to a QTRAP 6500 + mass spectrometer (Sciex, Framingham, MA, United States). Chromatographic separation was achieved on a Thermo Accucore C_30_ column (100 mm × 2.1 mm, 2.6 μm; Thermo Fisher Scientific, Waltham, MA, United States) kept at 45 °C. Two mobile phases were used. Phase A was acetonitrile/water (60:40, v/v) with 0.1% formic acid and 10 mM ammonium formate. Phase B was acetonitrile/isopropanol (10:90, v/v) with 0.1% formic acid and 10 mM ammonium formate. The flow rate was set to 0.35 mL/min, and the injection volume was set to 2 μL. Mass spectrometric detection was performed with an electrospray ionization (ESI) source operated in both positive and negative ion modes. The source temperature was set to 500 °C, and the ion spray voltage was set to +5500 V in positive mode and −5,500 V in negative mode. The pressures for ion source gas 1 (GS1), gas 2 (GS2), and curtain gas (CUR) were set to 45, 55, and 35 psi, respectively. Data were acquired in multiple reaction monitoring (MRM) mode, with declustering potentials and collision energies optimized for each transition. Lipid standards were obtained from Sigma-Aldrich (St. Louis, MO, United States) and Avanti Polar Lipids (Alabaster, AL, United States).

### Stable heterologous overexpression in tobacco

For tobacco (*N. tabacum*) transformation, the full-length CDS of *PpGELP18* was inserted into the vector pCAMBIA1301 and transformed into *Agrobacterium tumefaciens* strain EHA105. The leaf disc co-cultivation method (Horsch et al. 1985) was used to perform transformation in tobacco (*N. tabacum*). Transgenic plants were screened on selected medium containing 10 mg/L hygromycin B (HygB) and kept in a growth room (16 h light and 8 h dark, 25 °C).

### Chilling stress treatment and chilling tolerance evaluation

Chilling stress treatment was conducted as previously described (Ai et al. 2025). Surface-sterilized seeds from T_0_ transgenic and WT tobacco (*N. tabacum*) were sown on Murashige and Skoog (MS) medium. The medium for transgenic seeds was supplemented with 10 mg/L hygromycin B for selection, while the WT seeds were grown on antibiotic-free MS medium. After 21 d, half the plants were transferred to 0 °C for chilling treatment (16 h light and 8 h dark) for 14 d, while the other half, served as a control, were immediately assayed. The maximum photochemical efficiency of photosystem II (Fv/Fm) was determined using an Imaging-PAM Chlorophyll Fluorometer equipped with a computer-operated PAM control unit (IMAG-MAXI; Heinz Walz, Effeltrich, Germany) following the method described previously (Guo et al. 2024). Leaf samples were snap-frozen in liquid nitrogen and stored at −80 °C for subsequent assays.

### Subcellular localization assay

The coding sequences of *PpGELP18,* without the stop codon, were cloned into the pCAMBIA2300-eGFP vector and introduced into *A. tumefaciens* strain GV3101. The bacteria were infiltrated into mCherry-NLS transgenic *N*. *benthamiana* leaves using our previous protocols (Liang et al. 2024). Fluorescence images were taken with confocal laser scanning (Nikon A1-SHS, Tokyo, Japan) 2 d after infiltration.

### Bioinformatic analysis of protein sequences and enrichment analysis

*In silico* analysis of PpGELP18 conserved domains was performed using the National Center for Biotechnology Information (NCBI) CD-Search (https://www.ncbi.nlm.nih.gov/Structure/cdd/wrpsb.cgi) and InterPro (https://www.ebi.ac.uk/interpro/search/sequence/). The transmembrane domain prediction of PpGELP18 was performed using DeepTMHMM 1.0 (https://services.healthtech.dtu.dk/services/DeepTMHMM-1.0/). A multiple sequence alignment based on amino acid sequences between PpGELP18 and AtGELP33 was performed using DNAMAN. For phylogenetic analysis, amino acid sequences of AtGELP family members from *A. thaliana* were acquired from The Arabidopsis Information Resource (TAIR; https://www.arabidopsis.org/). The corresponding sequences for peach (*P. persica*) were retrieved from the Genome Database for Rosaceae (GDR; https://www.rosaceae.org/). Multiple sequence alignment and phylogenetic tree construction were performed using MEGA version 12. A phylogenetic tree was inferred using the Neighbor-Joining (NJ) method. The robustness of the inferred tree topology was evaluated by bootstrap analysis with 1,000 replicates. The resulting tree was visualized and annotated using the Interactive Tree of Life (iTOL, https://itol.embl.de) online tool. For functional annotation and pathway mapping, the protein sequences were subjected to a BLAST search against the KEGG database (https://www.kegg.jp/) to obtain their KEGG Orthology (KO) identifiers. These identifiers were then mapped to specific pathways within the KEGG system.

### Statistical analysis

The mean ± standard error from 3 independent biological replicates was presented in this study. Data analysis was performed using SPSS 23.0 (IBM Corp., Armonk, N.Y., United States) by one-way analysis of variance (ANOVA) and unpaired 2-sample Student's *t*-test. Figures were drawn using GraphPad Prism 9.0.2 (GraphPad Software, San Diego, CA, United States), OriginPro 2026 (OriginLab Corporation, Northampton, MA, United States) and TBtools (Chen et al. 2023).

## Supplementary Material

kiag705_Supplementary_Data

## Data Availability

All data included in this study are available within the article and its supplementary materials.

## References

[kiag705-B1] Ai SJ et al 2025. PpERF17 alleviates peach fruit postharvest chilling injury under elevated CO_2_ by activating jasmonic acid and γ-aminobutyric acid biosynthesis. Hortic Res. 12:uhaf014. 10.1093/hr/uhaf014. PMC11908827 40093381

[kiag705-B2] Akoh CC, Lee G-C, Liaw Y-C, Huang T-H, Shaw J-F. 2004. GDSL family of serine esterases/lipases. Prog Lipid Res. 43:534–552. 10.1016/j.plipres.2004.09.002. 15522763

[kiag705-B3] Almeida AM et al 2016. Proteomic analysis of a segregant population reveals candidate proteins linked to mealiness in peach. J Proteomics. 131:71–81. 10.1016/j.jprot.2015.10.011. 26459401

[kiag705-B4] Barbaglia AM, Tamot B, Greve V, Hoffmann-Benning S. 2016. Phloem proteomics reveals new lipid-binding proteins with a putative role in lipid-mediated signaling. Front Plant Sci. 7:563. 10.3389/fpls.2016.00563. PMC4849433 27200036

[kiag705-B5] Baudouin E et al 1997. Functional expression of a tobacco gene related to the serine hydrolase family -- esterase activity towards short-chain dinitrophenyl acylesters. Eur J Biochem. 248:700–706. 10.1111/j.1432-1033.1997.t01-1-00700.x. 9342220

[kiag705-B6] Bensimon A, Heck AJR, Aebersold R. 2012. Mass spectrometry-based proteomics and network biology. Annu Rev Biochem. 81:379–405. 10.1146/annurev-biochem-072909-100424. 22439968

[kiag705-B7] Browse J, Somerville CR. 1994. Glycerolipids. In: Meyerowitz EM, Somerville CR, editors. Arabidopsis. Cold Spring Harbor Laboratory Press. p. 881–936.

[kiag705-B8] Cao YP et al 2018. Expansion and evolutionary patterns of GDSL-type esterases/lipases in Rosaceae genomes. Funct Integr Genomics. 18:673–684. 10.1007/s10142-018-0620-1. 29948460

[kiag705-B9] Chen CJ et al 2023. TBtools-II: a “one for all, all for one” bioinformatics platform for biological big-data mining. Mol Plant. 16:1733–1742. 10.1016/j.molp.2023.09.010. 37740491

[kiag705-B10] de Almeida CP, Barbosa RR, Ferraz CG, de Castro RD, Ribeiro PR. 2024. Genome-wide identification of the GDSL-type esterase/lipase protein (GELP) gene family in *Ricinus communis* and its transcriptional regulation during germination and seedling establishment under different abiotic stresses. Plant Physiol Biochem. 214:108939. 10.1016/j.plaphy.2024.108939. 39029309

[kiag705-B11] Ding L-N et al 2019a. Improving seed germination and oil contents by regulating the GDSL transcriptional level in *Brassica napus*. Plant Cell Rep. 38:243–253. 10.1007/s00299-018-2365-7. 30535511

[kiag705-B12] Ding L-N et al 2019b. Advances in plant GDSL lipases: from sequences to functional mechanisms. Acta Physiol Plant. 41:151. 10.1007/s11738-019-2944-4.

[kiag705-B13] Ding L-N et al 2024a. A GDSL motif-containing lipase modulates *Sclerotinia sclerotiorum* resistance in *Brassica napus*. Plant Physiol. 196:2973–2988. 10.1093/plphys/kiae500. PMC11638095 39321167

[kiag705-B14] Ding YL, Shi YT, Yang SH. 2024b. Regulatory networks underlying plant responses and adaptation to cold stress. Annu Rev Genet. 58:43–65. 10.1146/annurev-genet-111523-102226. 39018466

[kiag705-B15] Ernst R, Ejsing CS, Antonny B. 2016. Homeoviscous adaptation and the regulation of membrane lipids. J Mol Biol. 428:4776–4791. 10.1016/j.jmb.2016.08.013. 27534816

[kiag705-B16] Fruk G, Cmelik Z, Jemric T, Hribar J, Vidrih R. 2014. Pectin role in woolliness development in peaches and nectarines: a review. Sci Hortic. 180:1–5. 10.1016/j.scienta.2014.09.042.

[kiag705-B17] Gao L et al 2024. Genetic and lipidomic analyses reveal the key role of lipid metabolism for cold tolerance in maize. J Genet Genomics. 51:326–337. 10.1016/j.jgg.2023.07.004. 37481121

[kiag705-B18] Gao MJ et al 2017. GDSL lipases modulate immunity through lipid homeostasis in rice. PLoS Pathog. 13:e1006724. 10.1371/journal.ppat.1006724. PMC5703576 29131851

[kiag705-B19] Gilmour SJ, Fowler SG, Thomashow MF. 2004. Arabidopsis transcriptional activators CBF1, CBF2, and CBF3 have matching functional activities. Plant Mol Biol. 54:767–781. 10.1023/B:PLAN.0000040902.06881.d4. 15356394

[kiag705-B20] Guo MY et al 2024. Loss of cold tolerance is conferred by absence of the WRKY34 promoter fragment during tomato evolution. Nat Commun. 15:6667. 10.1038/s41467-024-51036-y. PMC11303406 39107290

[kiag705-B21] Guo XO, Xu JJ, Cui XH, Chen H, Qi HY. 2017. iTRAQ-based protein profiling and fruit quality changes at different development stages of oriental melon. BMC Plant Biol. 17:28. 10.1186/s12870-017-0977-7. PMC5273850 28129739

[kiag705-B22] Harayama T, Riezman H. 2018. Understanding the diversity of membrane lipid composition. Nat Rev Mol Cell Biol. 19:281–296. 10.1038/nrm.2017.138. 29410529

[kiag705-B23] Horsch RB et al 1985. A simple and general method for transferring genes into plants. Science. 227:1229–1231. 10.1126/science.227.4691.1229. 17757866

[kiag705-B24] Hou YY et al 2022. CaCl_2_ mitigates chilling injury in loquat fruit via the CAMTA5-mediated transcriptional repression of membrane lipid degradation genes. Food Res Int. 162:111966. 10.1016/j.foodres.2022.111966. 36461216

[kiag705-B25] Hu HH et al 2008. Characterization of transcription factor gene *SNAC2* conferring cold and salt tolerance in rice. Plant Mol Biol. 67:169–181. 10.1007/s11103-008-9309-5. 18273684

[kiag705-B26] Hu SQ et al 2022. 24-Epibrassinolide improves chilling tolerance by regulating PpCBF5-mediated membrane lipid metabolism in peach fruit. Postharvest Biol Technol. 186:111844. 10.1016/j.postharvbio.2022.111844.

[kiag705-B27] Huang D et al 2024. PpBBX32 and PpZAT5 modulate temperature-dependent and tissue-specific anthocyanin accumulation in peach fruit. Hortic Res. 11:uhae212. 10.1093/hr/uhae212. PMC11462610 39385999

[kiag705-B28] Ishikawa HA . 1996. Ultrastructural features of chilling injury: injured cells and the early events during chilling of suspension-cultured mung bean cells. Am J Bot. 83:825–835. 10.1002/j.1537-2197.1996.tb12774.x.

[kiag705-B29] Jiao CF . 2021. IP3 mediates NO-enhanced chilling tolerance in postharvest kiwifruit. Postharvest Biol Technol. 176:111463. 10.1016/j.postharvbio.2021.111463.

[kiag705-B30] Jung J-H, Seo PJ, Oh E, Kim J. 2023. Temperature perception by plants. Trends Plant Sci. 28:924–940. 10.1016/j.tplants.2023.03.006. 37045740

[kiag705-B31] Kwon SJ et al 2009. GDSL lipase-like 1 regulates systemic resistance associated with ethylene signaling in *Arabidopsis*. Plant J. 58:235–245. 10.1111/j.1365-313X.2008.03772.x. 19077166

[kiag705-B32] Liang L et al 2024. Molecular mechanisms underlying natural deficient and ultraviolet-induced accumulation of anthocyanin in the peel of ‘Jinxiu’ peach. Plant Cell Environ. 47:4833–4848. 10.1111/pce.15064. 39101482

[kiag705-B33] Liu DF et al 2024. The GDSL lipase CpGLIP1 from *Chimonanthus praecox* improves drought and cold tolerance in *Arabidopsis* and poplar. Ind Crops Prod. 215:118636. 10.1016/j.indcrop.2024.118636.

[kiag705-B34] Liu MJ et al 2025. Flexible regulation of photosynthesis to low temperature in alfalfa contributed to spring rejuvenation. Plant Physiol Biochem. 229:110501. 10.1016/j.plaphy.2025.110501. 40953536

[kiag705-B35] Liu Y, Beyer A, Aebersold R. 2016. On the dependency of cellular protein levels on mRNA abundance. Cell. 165:535–550. 10.1016/j.cell.2016.03.014. 27104977

[kiag705-B36] Lurie S . 2021. Genomic and transcriptomic studies on chilling injury in peach and nectarine. Postharvest Biol Technol. 174:111444. 10.1016/j.postharvbio.2020.111444.

[kiag705-B37] Lurie S, Crisosto CH. 2005. Chilling injury in peach and nectarine. Postharvest Biol Technol. 37:195–208. 10.1016/j.postharvbio.2005.04.012.

[kiag705-B38] Luza JG, Rv G, Polito VS, Kader AA. 1992. Chilling injury in peaches: a cytochemical and ultrastructural cell wall study. J Amer Soc Hort Sci. 117:114–118. 10.21273/JASHS.117.1.114.

[kiag705-B39] Mawson BT, Cummins WR. 1991. Low temperature acclimation of guard cell chloroplasts by the Arctic plant *Saxifraga cernua* L. Plant Cell Environ. 14:569–576. 10.1111/j.1365-3040.1991.tb01527.x.

[kiag705-B40] Momo J et al 2023. Proteomics of reproductive development, fruit ripening, and stress responses in tomato. J Agric Food Chem. 71:65–95. 10.1021/acs.jafc.2c06564. 36584279

[kiag705-B41] Musser RL, Thomas SA, Wise RR, Peeler TC, Naylor AW. 1984. Chloroplast ultrastructure, chlorophyll fluorescence, and pigment composition in chilling-stressed soybeans. Plant Physiol. 74:749–754. 10.1104/pp.74.4.749. PMC1066762 16663504

[kiag705-B42] Nilo R et al 2010. Proteomic analysis of peach fruit mesocarp softening and chilling injury using difference gel electrophoresis (DIGE). BMC Genomics. 11:43. 10.1186/1471-2164-11-43. PMC2822761 20082721

[kiag705-B43] Ning M et al 2022. Low-temperature adaptation and preservation revealed by changes in physiological-biochemical characteristics and proteome expression patterns in post-harvest Hami melon during cold storage. Planta. 255:91. 10.1007/s00425-022-03874-7. 35320421

[kiag705-B44] Peppino Margutti M et al 2018. Differential phosphatidic acid metabolism in barley leaves and roots induced by chilling temperature. Plant Physiol Biochem. 132:174–182. 10.1016/j.plaphy.2018.08.038. 30199789

[kiag705-B45] Rodrigues M et al 2024. Dissecting postharvest chilling injuries in pome and stone fruit through integrated omics. Front Plant Sci. 14:1272986. 10.3389/fpls.2023.1272986. PMC10791837 38235207

[kiag705-B46] Ruiz-Lopez N et al 2021. Synaptotagmins at the endoplasmic reticulum-plasma membrane contact sites maintain diacylglycerol homeostasis during abiotic stress. Plant Cell. 33:2431–2453. 10.1093/plcell/koab122. PMC8364230 33944955

[kiag705-B47] Salazar-Salas NY et al 2022. Proteomic changes in mango fruit peel associated with chilling injury tolerance induced by quarantine hot water treatment. Postharvest Biol Technol. 186:111838. 10.1016/j.postharvbio.2022.111838.

[kiag705-B48] Shomo ZD, Li F, Smith CN, Edmonds SR, Roston RL. 2024. From sensing to acclimation: the role of membrane lipid remodeling in plant responses to low temperatures. Plant Physiol. 196:1737–1757. 10.1093/plphys/kiae382. 39028871

[kiag705-B49] Song CB et al 2022. NAC-mediated membrane lipid remodeling negatively regulates fruit cold tolerance. Hortic Res. 9:uhac039. 10.1093/hr/uhac039. PMC9071380 35531317

[kiag705-B50] Su R et al 2024. GDSL lipase gene *HTA1* negatively regulates heat tolerance in rice seedlings by regulating reactive oxygen species accumulation. Antioxidants. 13:592. 10.3390/antiox13050592. PMC11117967 38790697

[kiag705-B51] Tan JJ, Feng HQ, Deng ZP. 2025. DIA-based phosphoproteomics profiling reveals rapid cold-responsive phosphorylation events in tomato fruit. Postharvest Biol Technol. 219:113207. 10.1016/j.postharvbio.2024.113207.

[kiag705-B52] Upton C, Buckley JT. 1995. A new family of lipolytic enzymes? Trends Biochem Sci. 20:178–179. 10.1016/S0968-0004(00)89002-7. 7610479

[kiag705-B53] Ursache R et al 2021. GDSL-domain proteins have key roles in suberin polymerization and degradation. Nat Plants. 7:353–364. 10.1038/s41477-021-00862-9. PMC7610369 33686223

[kiag705-B54] Vogel C, Marcotte EM. 2012. Insights into the regulation of protein abundance from proteomic and transcriptomic analyses. Nat Rev Genet. 13:227–232. 10.1038/nrg3185. PMC3654667 22411467

[kiag705-B55] Wang HW et al 2024. Multi-omics analysis of the effects of low-temperature storage on chilling injury of bitter gourd. Postharvest Biol Technol. 218:113161. 10.1016/j.postharvbio.2024.113161.

[kiag705-B56] Wang K et al 2017. Transcriptomic and metabolic analyses provide new insights into chilling injury in peach fruit. Plant Cell Environ. 40:1531–1551. 10.1111/pce.12951. 28337785

[kiag705-B57] Welti R et al 2002. Profiling membrane lipids in plant stress responses. J Biol Chem. 277:31994–32002. 10.1074/jbc.M205375200. 12077151

[kiag705-B58] Wise RR, McWilliam JR, Naylor AW. 1983. A comparative study of low-temperature-induced ultrastructural alterations of three species with differing chilling sensitivities. Plant Cell Environ. 6:525–535. 10.1111/j.1365-3040.1983.tb01165.x.

[kiag705-B59] Wise RR, Naylor AW. 1987. Chilling-enhanced photooxidation: the peroxidative destruction of lipids during chilling injury to photosynthesis and ultrastructure. Plant Physiol. 83:272–277. 10.1104/pp.83.2.272. PMC1056347 16665235

[kiag705-B60] Xu DY et al 2023a. A comparative proteomic and metabolomic analysis of the low-temperature response of a chilling-injury sensitive and a chilling-injury tolerant cultivar of green bell pepper. Sci Hortic. 318:112092. 10.1016/j.scienta.2023.112092.

[kiag705-B61] Xu P et al 2023b. Amelioration of chilling injury in ‘Guifei’ mango fruit by melatonin is associated with regulation of lipid metabolic enzymes and remodeling of lipidome. Postharvest Biol Technol. 198:112233. 10.1016/j.postharvbio.2022.112233.

[kiag705-B62] Yamazaki T, Kawamura Y, Minami A, Uemura M. 2008. Calcium-dependent freezing tolerance in *Arabidopsis* involves membrane resealing via synaptotagmin SYT1. Plant Cell. 20:3389–3404. 10.1105/tpc.108.062679. PMC2630449 19088330

[kiag705-B63] Yan XX et al 2024. Cloning and functional study of *AmGDSL1* in *Agropyron mongolicum*. Int J Mol Sci. 25:9467. 10.3390/ijms25179467. PMC11395167 39273413

[kiag705-B64] Yin GK et al 2009. Mitochondrial damage in the soybean seed axis during imbibition at chilling temperatures. Plant Cell Physiol. 50:1305–1318. 10.1093/pcp/pcp074. 19520672

[kiag705-B65] Yuan JW et al 2025. Genome-wide identification and evolution-profiling analysis of the GDSL gene family in apple (*Malus* × *domestica*). Agronomy. 15:517. 10.3390/agronomy15030517.

[kiag705-B66] Zhai XL et al 2025. Genome-wide identification of the GDSL esterase/lipase gene family in cucumber and functional characterization of *CsGELP57* in fruit cuticle development. Hortic Plant J. 11:2289–2291. 10.1016/j.hpj.2025.06.010.

[kiag705-B67] Zhang PJ, Zhang H, Du JK, Qiao YS. 2022. Genome-wide identification and co-expression analysis of GDSL genes related to suberin formation during fruit russeting in pear. Hortic Plant J. 8:153–170. 10.1016/j.hpj.2021.11.010.

[kiag705-B68] Zhao H et al 2020. The GDSL lipase MHZ11 modulates ethylene signaling in rice roots. Plant Cell. 32:1626–1643. 10.1105/tpc.19.00840. PMC7203933 32184349

[kiag705-B69] Zheng GW, Li LX, Li WQ. 2016. Glycerolipidome responses to freezing- and chilling-induced injuries: examples in *Arabidopsis* and rice. BMC Plant Biol. 16:70. 10.1186/s12870-016-0758-8. PMC4802656 27000868

[kiag705-B70] Zhou HJ et al 2022. Controlled atmosphere storage alleviates internal browning in flat peach fruit by regulating energy and sugar metabolisms. Plant Physiol Biochem. 186:107–120. 10.1016/j.plaphy.2022.07.003. 35835077

[kiag705-B71] Zhu YC et al 2019. Effect of ethylene on cell wall and lipid metabolism during alleviation of postharvest chilling injury in peach. Cells. 8:1612. 10.3390/cells8121612. PMC6952997 31835827

[kiag705-B72] Zhu Y-C et al 2020. DNA demethylation is involved in the regulation of temperature-dependent anthocyanin accumulation in peach. Plant J. 102:965–976. 10.1111/tpj.14680. 31923329

[kiag705-B73] Zhu YC et al 2021. DNA hypermethylation associated with the development of temperature-dependent postharvest chilling injury in peach fruit. Postharvest Biol Technol. 181:111645. 10.1016/j.postharvbio.2021.111645.

