# Supplementary material for: A peach esterase/lipase mediates lipid remodeling as a protective mechanism against chilling injury
Source: Plant Physiol. 2026 Sep 21;202(2):kiag705. doi: 10.1093/plphys/kiag705 (PMC13651905; doi:10.1093/plphys/kiag705)
Supplement: Supplementary file 1 [file kiag705_supplementary_data.zip › Supplementary Figure R2.docx]

**Supplemental Figures**

**Supplemental Figure S1.** Kyoto Encyclopedia of Genes and Genomes (KEGG) enrichment analysis of differentially accumulated proteins (DAPs) from peach fruit stored at different temperatures for 40 d. The top 20 pathways, ranked by DAP count, are shown.

**Supplemental Figure S2.** Kyoto Encyclopedia of Genes and Genomes (KEGG) enrichment analysis of differentially accumulated proteins (DAPs) from peach fruit stored at 5 °C for different durations. The top 20 pathways, ranked by DAP count, are shown.

**Supplemental Figure S3.** Protein abundance of M5Y1K8 in peach fruit stored at different temperatures for 40 d (A) and after different storage durations at 5 °C (B). Mean ± SE from three independent biological replicates were presented. Different lowercase letters denoted a significant difference (*P* < 0.05).

**Supplemental Figure S4.** Conserved domains of M5Y1K8 as revealed via *in silico* analysis by InterPro (https://www.ebi.ac.uk/interpro/search/sequence/).

**Supplemental Figure S5.** Phylogenetic analysis of PpGELPs and AtGELPs.

**Supplemental Figure S6.** Expression and purification of glutathione *S*-transferase (GST) protein (A) and recombinant GST-PpGELP18 protein (B) in *E. coli*. Proteins were separated by sodium dodecyl sulfate – polyacrylamide gel electrophoresis (SDS-PAGE) and visualized by Coomassie Brilliant Blue R-250 staining. Lane 1, total cell lysate; Lane 2, flow-through; Lane 3-7, the first five elution fractions collected during affinity chromatography. The arrow indicates the target protein band of expected size ~26 kDa for GST and ~68 kDa for recombinant GST-PpGELP18 protein.

**Supplemental Figure S7.** Expression levels of *PpGELP18* in wild-type (WT) and transgenic tobacco (*N. tabacum*) plants subjected to 0 °C treatment. Data are presented as means of three biological replicates ± SE. Asterisks indicate statistically significant differences compared with the WT (* *P* < 0.05; ** *P* < 0.01).

**Supplemental** **Figure S8.** Principal component analysis (PCA) of lipidomics data from low temperature treated wild-type (WT) and transgenic tobacco (*N. tabacum*) plants. (A) PCA score plot illustrating the sample distribution of WT and transgenic lines; (B) PCA loading plot displaying the contribution of individual lipid subclass to the observed sample separation. ADGGA, acyl diacylglyceryl glucuronide; CoQ, coenzyme Q (ubiquinone); DGCC, diacylglyceryl carboxyhydroxymethylcholine; DGGA, diacylglyceryl glucuronide; DGTS, diacylglyceryl-*N*,*N*,*N*-trimethylhomoserine; PMeOH, phosphatidylmethanol. The other abbreviations for lipid compounds are same as listed in Figure 5.

**Supplemental Figure S9.** Heatmap visualization of lipid species that were differentially accumulated between wild-type (WT) and transgenic tobacco (*N. tabacum*) plants after low-temperature treatment. Each row represents an individual lipid species, and colors indicate the relative abundance (yellow, increased; green, decreased). Lipid species in red text: lipid species up-regulated in transgenic plants; Lipid species in green text: lipid species down-regulated in transgenic plants. Asterisks (*, **, ***) indicate that mean values are significantly different between transgenic plant and WT (*P* < 0.05, *P* < 0.01, *P* < 0.001) according to Duncan’s multiple range test. The abbreviations for lipid compounds are same as listed in Figure 5.

**Supplemental Figure S10.** Heatmap visualization of triacylglycerol (TAG) species that were differentially accumulated between wild-type (WT) and transgenic tobacco (*N. tabacum*) plants after low-temperature treatment. Each row represents an individual lipid species, and colors indicate the relative abundance (yellow, increased; green, decreased). Lipid species in red text: lipid species up-regulated in transgenic plants; Lipid species in green text: lipid species down-regulated in transgenic plants. Asterisks (*, **, ***) indicate that mean values are significantly different between transgenic plant and WT (*P* < 0.05, *P* < 0.01, *P* < 0.001) according to Duncan’s multiple range test.

**Supplemental Figure S11.** Representative transmission electron microscopy (TEM) images of wild-type (WT) and transgenic tobacco (*N. tabacum*) mesophyll cells before (control, 25 °C) and after chilling treatment (cold, 0 °C). Left column: Overview showing an intact central vacuole, with organelles confined to the cell periphery. Middle column: Detailed view of chloroplasts. Right column: Detailed view of mitochondria. Note: Scale bars vary to show detail and context. Chlo, Chloroplast; Cris, Cristae; CW, Cell wall; GL, Granum lamellae; Mito, Mitochondrion; SG, Starch granule.

**Supplemental Figure S12.** Photos of peach fruit after 3 days at 20 °C following 0, 5, 8, 12, 16 °C storage for 40 d. White bar: 5 cm.
